# Cytosine base editors induce off-target mutations and adverse phenotypic effects in transgenic mice

**DOI:** 10.1038/s41467-023-37508-7

**Published:** 2023-03-30

**Authors:** Nana Yan, Hu Feng, Yongsen Sun, Ying Xin, Haihang Zhang, Hongjiang Lu, Jitan Zheng, Chenfei He, Zhenrui Zuo, Tanglong Yuan, Nana Li, Long Xie, Wu Wei, Yidi Sun, Erwei Zuo

**Affiliations:** 1 Shenzhen Branch, Guangdong Laboratory for Lingnan Modern Agriculture, Key Laboratory of Synthetic Biology, Ministry of Agriculture and Rural Affairs, Agricultural Genomics Institute at Shenzhen, Chinese Academy of Agricultural Sciences, Shenzhen, China; 2 Key Laboratory of Agricultural Animal Genetics, Breeding and Reproduction of Ministry of Education & Key Lab of Swine Genetics and Breeding of Ministry of Agriculture and Rural Affairs, Huazhong Agricultural University, Wuhan, China; 3 State Key Laboratory for Conservation and Utilization of Subtropical Agro-Bioresources, College of Animal Science and Technology, Guangxi University, Nanning, China; 4 CAS Key Laboratory of Computational Biology, Shanghai Institute of Nutrition and Health, University of Chinese Academy of Sciences, Chinese Academy of Sciences, Shanghai, China; 5 Lingang Laboratory, Shanghai, China; 6 Institute of Neuroscience, CAS Center for Excellence in Brain Science and Intelligence Technology, Chinese Academy of Sciences, Shanghai, China

**Keywords:** Genetic engineering, CRISPR-Cas9 genome editing

## Abstract

Base editors have been reported to induce off-target mutations in cultured cells, mouse embryos and rice, but their long-term effects in vivo remain unknown. Here, we develop a Systematic evaluation Approach For gene Editing tools by Transgenic mIce (SAFETI), and evaluate the off-target effects of BE3, high fidelity version of CBE (YE1-BE3-FNLS) and ABE (ABE7.10^F148A^) in ~400 transgenic mice over 15 months. Whole-genome sequence analysis reveals BE3 expression generated de novo mutations in the offspring of transgenic mice. RNA-seq analysis reveals both BE3 and YE1-BE3-FNLS induce transcriptome-wide SNVs, and the numbers of RNA SNVs are positively correlated with CBE expression levels across various tissues. By contrast, ABE7.10^F148A^ shows no detectable off-target DNA or RNA SNVs. Notably, we observe abnormal phenotypes including obesity and developmental delay in mice with permanent genomic BE3 overexpression during long-time monitoring, elucidating a potentially overlooked aspect of side effects of BE3 in vivo.

## Abstract (web summary)

The potential off-target effects of long-term expression of base editors in vivo are unclear. Here the authors report SAFETI, Systematic evaluation Approach For gene Editing tools by Transgenic mIce, to examine off-target effects of base editors over time in mice, and see abnormal side effects.

## Introduction

CRISPR-derived base editing is a genome editing method to introduce point mutations on DNA or RNA at the target loci. By fusing catalytically dead Cas9 (dCas9) or nickase Cas9 (nCas9) with cytidine deaminases or adenosine deaminases, cytosine base editors (CBEs)^1,2^ and adenine base editors (ABEs)^3^ were developed to install targeted C-to-T or A-to-G point mutations without generating double-strand breaks (DSBs). However, the application of CBEs and ABEs was limited by off-target DNA and RNA mutations^4–7^. Even though high-fidelity base editors were subsequently generated by protein engineering^4,8–10^, their specificity in vivo remains to be explored considering their constant and long-term expression through common delivery strategies^11,12^.

Here we generate transgenic mouse lines expressing BE3, high fidelity YE1-BE3-FNLS (W90Y and R126E mutations in rAPOEBC1)^8^, or ABE7.10^F148A^ (F148A in TadA)^4^, and comprehensively evaluate the side effects of base editors on DNA and RNA across various tissues. In addition, we also monitor the phenotypes of the transgenic mice over months to explore the adverse effects of long-term expression of BEs.

## Results

### Generation of transgenic mice expressing base editors

**Fig. 1:**
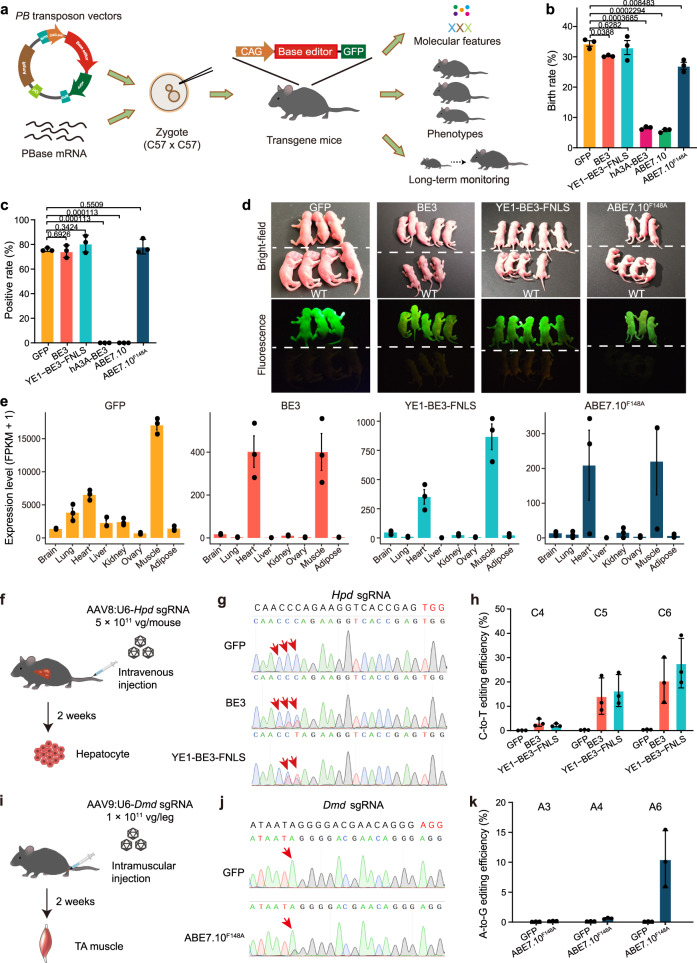
Generation of GFP, BE3, YE1-BE3-FNLS, and ABE7.10^F148A^ mouse lines. **a** Schematic diagram of the Systematic evaluation Approach For gene Editing tools by Transgenic mIce (SAFETI). **b** Birth rate of F0 mice injected with GFP, BE3, hA3A-BE3, YE1-BE3-FNLS, ABE7.10 or ABE7.10^F148A^. Birth rate: the percentage of number of live births divided by the population size of transferred embryos. The birth rates were calculated for three injections, and the total numbers of transferred embryos were 265, 258, 259, 320, 302 and 265 in GFP, BE3, hA3A-BE3, YE1-BE3-FNLS, ABE7.10 and ABE7.10^F148A^, respectively. **c** The proportion of F0 mice with successful integration of the transgenes (positive rate). The positive rates were calculated for three transplantations, and the total number of births were 91, 78, 86, 21, 17 and 72 in GFP, BE3, YE1-BE3-FNLS, hA3A-BE3, ABE7.10 and ABE7.10^F148A^, respectively. **d** Bright-field and fluorescence images of newborn wild-type (WT) mice and mice expressing GFP, BE3, YE1-BE3-FNLS, or ABE7.10^F148A^. **e** The expression levels of transgenes in the indicated tissues. FPKM, Fragments Per Kilobase Million. **f** Schematic showing the experimental procedure for delivery of *Hpd* sgRNA through AAV8 into the CBE transgenic mice by tail-vein injection. **g** Representative Sanger sequencing chromatograms of PCR amplicons spanning the *Hpd* gRNA target site are shown for GFP, BE3, and YE1-BE3-FNLS transgenic mice. Red arrows mark the targeted cytosine. **h** The C-to-T base editing efficiency at the *Hpd* target site in GFP, BE3, and YE1-BE3-FNLS mice assessed by deep sequencing. **i** Schematic showing the experimental procedure for delivery of *Dmd* sgRNA through AAV9 into the ABE transgenic mice by intramuscular injection. **j** Representative Sanger sequencing chromatograms of PCR amplicons spanning the *Dmd* target site are shown for GFP and ABE7.10^F148A^ mice. Red arrows mark the targeted adenine. **k** The A-to-G base editing efficiency at the *Dmd* target site (A3, A4, A6) in GFP and ABE7.10^F148A^ mice assessed by deep sequencing. Data are presented as means ± SEM (*n* = 3 biologically independent samples). *P* values were calculated by two-sided, unpaired *t*-test. Source data are provided as a Source Data file.

We generated transgenic mouse lines expressing BE3^1^, YE1-BE3-FNLS^8^, hA3A-BE3^13^, ABE7.10^3^, or ABE7.10^F148A4^ using the *piggyBac* (*PB*) transposon integration system^14^ (Fig. 1a and Supplementary Fig. 1a). The transposon vectors consisted of a CAG promoter followed the sequence encoding one of the base editors that was linked (via a self-cleaving P2A peptide) to an enhanced green fluorescent protein reporter (eGFP; Supplementary Fig. 1a). The transposon vectors were injected into zygotes of the C57BL/6 J mice together with *PB* transposase enzyme (PBase) mRNA. The zygotes were developed to two-cell embryos in vitro, and then transferred into oviducts of pseudopregnant females of the ICR strain. We found that birth rates of F0 mice were not severely affected by injecting vectors consisting of BE3, YE1-BE3-FNLS or ABE7.10^F148A^ (Fig. 1b). Besides, fluorescence detection and PCR genotyping indicated that more than 75% of the born mice in these groups were successfully integrated with the corresponding base editor (Fig. 1c, Supplementary Table 1). By contrast, mice injected with vectors for BE3-human APOBEC3A (hA3A-BE3) or ABE7.10 showed dramatically reduced birth rates without successfully integrated pups (Fig. 1b, c), suggesting that hA3A-BE3 and ABE7.10 were toxic to embryos. F0 mice with the successful integration of the various base editors were intercrossed to establish transgenic mouse founders, with the cross-mating of founders yielding four transgenic mouse lines expressing GFP, BE3, YE1-BE3-FNLS, or ABE7.10^F148A^ (Fig. 1d). Analysis of transposon integration sites showed the transgenes were integrated into the intergenic or intronic regions at different locations across the mouse genome (Supplementary Fig. 1b, c, Supplementary Table 2). The average copy numbers in GFP, BE3, YE1-BE3-FNLS and ABE7.10^F148A^ transgenic mice were 3.3, 4.3, 3.7 and 2.3, respectively, no statistical difference was observed among different groups (Supplementary Fig. 1d, e).

We next performed RNA-seq analyses of eight tissues (brain, lung, heart, liver, kidney, ovary, muscle and adipose) from 8-week-old GFP, BE3, YE1-BE3-FNLS, and ABE7.10^F148A^ transgenic mice. The mRNA expression of all three base editors were highest in muscles, followed by the heart, and low in other tissues, and we noted that YE1-BE3-FNLS was expressed at higher levels than BE3 or ABE7.10^F148A^ across various tissues (Fig. 1e). Similar expression patterns of these base editors across various tissues were observed in immunoblotting of extracts from organs (Supplementary Fig. 1f). These findings are in line with previous reports of particularly high expression of transgenes in muscles and in the heart^15,16^, and may reflect the preferential activation of CAG promoter in these organs. Despite of the high expression of base editors, we observed no obvious morphological abnormalities in any of the examined organs of the 8-week-old transgenic mice (Supplementary Fig. 2a).

We next examined the on-target editing performance in vivo in the base editor (BE) transgenic mouse lines. For the BE3 and YE1-BE3-FNLS mouse lines, we constructed a U6-*Hpd* sgRNA vector containing an sgRNA for C-to-T editing of the *Hpd* locus (Supplementary Table 3), which results in a stop codon that can rescue the lethal phenotype of hereditary tyrosinemia type 1 in mice^17^. This vector was packaged into AAV2 serotype eight particles (AAV8) and delivered to 8-week-old CBE transgenic mice by tail-vein injection (Fig. 1f). Two weeks after delivery, we conducted Sanger and targeted deep sequencing to examine on-target editing in hepatocytes isolated from transgenic mice by FACS (Supplementary Fig. 2b) and detected successful C-to-T editing of the targeted site in ~20% and ~30% of the BE3 and YE1-BE3-FNLS hepatocytes, respectively (Fig. 1g, h, Supplementary Table 4).

We assessed A-to-G base editing activity of ABE7.10^F148A^ transgenic mice using an sgRNA targeting the *Dmd* gene (Supplementary Table 3), whose dysfunction results in Dunchenne muscular dystrophy (DMD)^18^. Specifically, the U6-*Dmd* sgRNA was packaged into AAV9 and delivered to the tibialis anterior (TA) muscle of 8-week-old ABE7.10^F148A^ mice by localized intramuscular injection^19^ (Fig. 1i). We collected TA muscles from 3 ABE7.10^F148A^ mice two weeks after delivery and found an average of 10.5% A-to-G editing at A6 of the targeted site by Sanger and targeted deep sequencing (Fig. 1j, k, Supplementary Table 4). Taken together, these results support that the genomically integrated BE3, YE1-BE3-FNLS, and ABE7.10^F148A^ of our transgenic mouse lines can successfully perform sgRNA-directed on-target editing in vivo.

### Base editors induced substantial genome-wide mutations in vivo

**Fig. 2:**
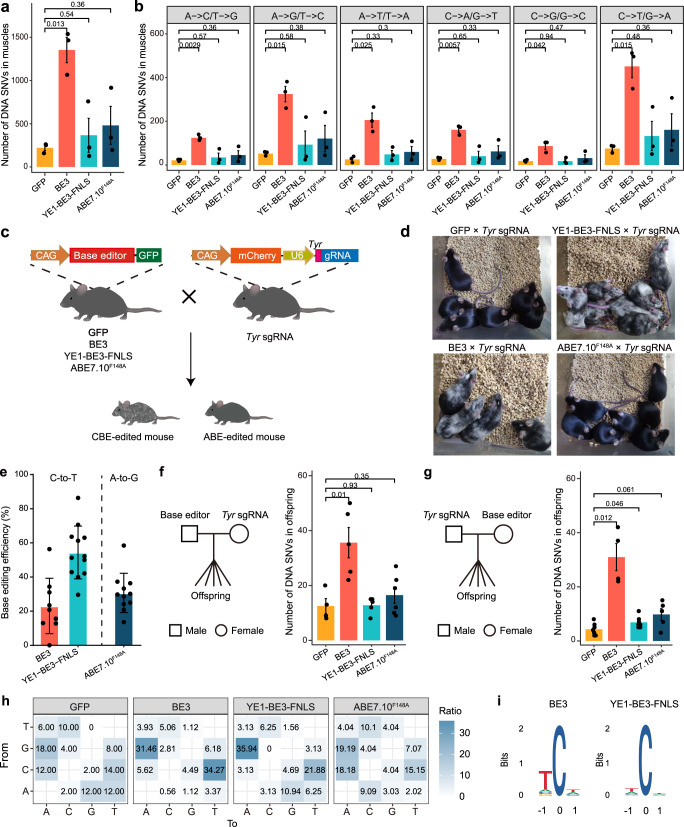
Off-target DNA mutations in the transgenic mice expressing base editors. **a** Comparison of the total number of DNA SNVs in muscles of GFP, BE3 YE1-BE3-FNLS and ABE7.10^F148A^ transgenic mice. *n* = 3 for each group. **b** Comparison of the number of DNA SNVs with the indicated mutation types in muscles of the GFP, BE3, YE1-BE3-FNLS, and ABE7.10^F148A^ transgenic mice. *n* = 3 for each group. **c** Schematic diagram for in vivo base editing verification by crossing *Tyr* sgRNA transgenic mice with GFP, BE3, YE1-BE3-FNLS, and ABE7.10^F148A^ mice. **d** Representative photos for the hair colors of offspring from GFP × *Tyr* sgRNA, BE3 × *Tyr* sgRNA, YE1-BE3-FNLS × *Tyr* sgRNA and ABE7.10^F148A^ × *Tyr* sgRNA mice. **e** The C-to-T or A-to-G base editing efficiency at *Tyr* target site in offspring of BE3 × *Tyr* sgRNA (*n* = 9), YE1-BE3-FNLS × *Tyr* sgRNA (*n* = 12) or ABE7.10^F148A^ × *Tyr* sgRNA (*n* = 11) breeding pairs. **f** Comparison of the number of de novo SNVs in offspring expressing GFP (*n* = 4), BE3 (*n* = 5), YE1-BE3-FNLS (*n* = 5) or ABE7.10^F148A^ (*n* = 6) with the *Tyr* sgRNA transgenic mice as mother. **g** Comparison of the number of de novo SNVs in offspring expressing GFP (*n* = 7), BE3 (*n* = 4), YE1-BE3-FNLS (*n* = 7) or ABE7.10^F148A^ (*n* = 5) with the *Tyr* sgRNA transgenic mice as father. *n* = biologically independent samples. Data are presented as mean ± SEM. **h** Distribution of mutation types of the de novo SNVs in offspring of crossing GFP/BE3/YE1-BE3-FNLS/ABE7.10^F148A^ father and *Tyr* sgRNA mother. The number indicates the percentage of a certain type of mutation among all mutations. **i** Sequence logos derived from the de novo SNVs in offspring of crossing BE3/YE1-BE3-FNLS father and *Tyr* sgRNA mother. Source data are provided as a Source Data file.

To evaluate whether the BE3, YE1-BE3-FNLS and ABE7.10^F148A^ editors introduce off-target edits in the transgenic mice, we performed whole genome sequencing (WGS) analysis of gDNA extracted from muscles (i.e., an organ with high base editor expression). To eliminate the influence of genetic background, we also performed WGS on the same tissues from GFP transgenic and wild-type (WT) mice. Mutations were called by three algorithms in the transgenic samples using the WT samples as the reference. Unlike few off-target mutations revealed by previous in vivo studies^19–23^, we found an average of 1353 SNVs in muscles of BE3 transgenic mice, which is 5 times higher than that in the GFP mice (Fig. 2a). These mutations were specifically observed in the transgenic mice rather than in the WT mice (Supplementary Fig. 3a). In addition, the numbers of C-to-T or G-to-A mutations were highest in the BE3 transgenic mice, and much higher than those in the GFP group (Fig. 2b, Supplementary Fig. 3b). This mutation bias was the same as that of cytosine deaminase rAPOBEC1^1^, suggesting these mutations were induced by BE3 expression. We also detected a significantly higher number of indels and structural variations (SVs) in the BE3 group than the GFP group (Supplementary Fig. 3c, d). By contrast, there were no differences in the numbers of SNVs or indels between the GFP and BE transgenic mice expressing high fidelity YE1-BE3-FNLS or ABE7.10^F148A^ (Fig. 2a, b and Supplementary Fig. 3c, d).

To further eliminate the interference of genetic background, we next examined the de novo off-target mutations induced by constant expression of base editors in 43 parent-offspring trios. We used the *PB* transposition system to generate another transgenic mouse line with a genomically integrated sgRNA cassette targeting the *Tyrosinase* (Tyr) gene for pigmentation^5,8^ (Fig. 2c and Supplementary Table 3). The targeted C-to-T editing by CBE directed by this sgRNA has been shown to introduce a stop codon at the *Tyr* gene that results in an Albino phenotype^5,8^. We then established four breeding pairs by crossing the GFP and BE transgenic male mice (BE3, YE1-BE3-FNLS or ABE7.10^F148A^) with *Tyr* sgRNA transgenic female mice, and 4 pairs of the reciprocal cross. The offspring of BE3 or YE1-BE3-FNLS × *Tyr* sgRNA mice showed black-white mosaic hair phenotypes (Fig. 2d), indicating the efficient editing of CBEs at the *Tyr* gene. We next performed WGS on gDNA extracted from tails of the progenies expressing both base editors and *Tyr* sgRNA, and confirmed the on-target editing efficiency of BE3 (26%), YE1-BE3-FNLS (54%), and ABE7.10^F148A^ (31%) at the *Tyr* locus (Fig. 2e).

To obtain the de novo generated mutations in the progenies, we performed WGS analysis on gDNA extracted from tails of 56 mice from 43 parent-offspring trios and called de novo variants in the offspring by filtering those shared with their parents or WT mice. Notably, we found that the number of de novo SNVs in the BE3 group was significantly higher than that in the GFP group (Fig. 2f, g and Supplementary Fig. 4a). The number of de novo SNVs in the YE1-BE3-FNLS and ABE7.10^F148A^ group was also slightly higher than that of the GFP samples when the BE transgenic mice were the mother (Fig. 2g). Interestingly, we noted that a higher number of de novo SNVs were observed in the offspring from crosses in which the BE transgenic mice were the father (Fig. 2f, g). A similar trend was observed in the GFP group, consistent with previous studies showing a paternal bias for de novo mutations^24^. The de novo mutations in the BE transgenic mice were evenly distributed across the chromosomes (Supplementary Fig. 4b).

Strikingly, the percentages of C-to-T and G-to-A mutations in the BE3 and YE1-BE3-FNLS transgenic mice were significantly higher than that of the GFP group (Fig. 2h and Supplementary Fig. 4c). Moreover, we found a consensus motif WCW (W = A or T) from the de novo mutations identified in the BE3 and YE1-BE3-FNLS transgenic mouse lines (Fig. 2i), consistent with the mutation preferences of the deaminase rAPOBEC1 in CBE^4,6^. Together, our results demonstrated that the expression of BE3 induces off-target DNA mutations in vivo, while the high fidelity YE1-BE3-FNLS and ABE7.10^F148A^ induces few off-target DNA mutations.

### Base editors induced transcriptome-wide mutations in vivo

**Fig. 3:**
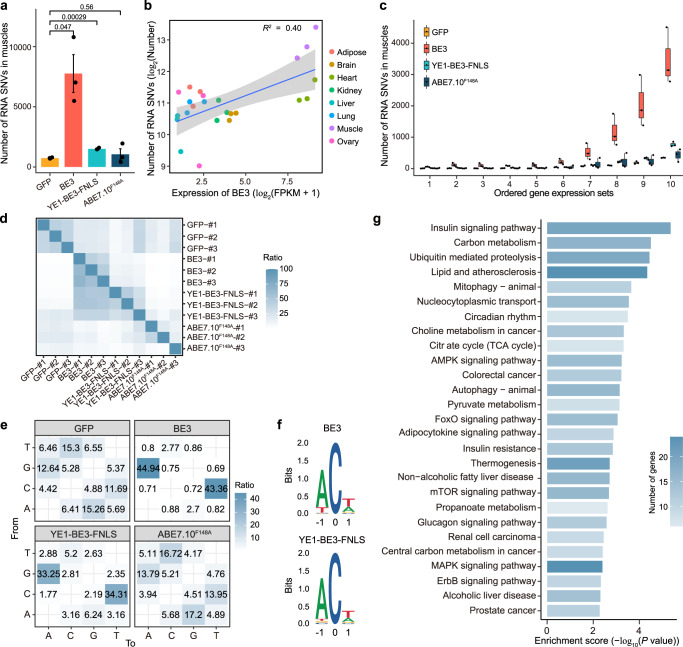
RNA off-target mutations in the GFP, BE3, YE1-BE3-FNLS and ABE7.10^F148A^ transgenic mice. **a** Comparison of the numbers of RNA SNVs in muscles of the GFP, BE3, YE1-BE3-FNLS and ABE7.10^F148A^ transgenic mice. Data are presented as mean ± SEM (*n* = 3). *P* values were calculated by two-sided unpaired *t*-test. **b** Correlations between the numbers of RNA SNVs and the expression levels of BE3 in diverse tissues of the BE3 transgenic mice. **c** Correlations between the numbers of RNA SNVs and gene expression levels in the muscles of GFP, BE3, YE1-BE3-FNLS, or ABE7.10^F148A^ transgenic mice. The gene expression levels were sorted by FPKM, and were equally divided into 10 sets in the order of expression levels. **d** The ratio of shared RNA SNVs between any two samples from GFP, BE3, YE1-BE3-FNLS, and ABE7.10^F148A^ groups. The proportion in each cell is calculated by the number of overlapping RNA SNVs between two samples divided by the number of RNA SNVs in the row. *n* = biologically independent samples. **e** Distribution of mutation types in muscles of the GFP, BE3, YE1-BE3-FNLS, and ABE7.10^F148A^ mice. The number indicates the percentage of a certain type of SNVs among all SNVs. **f** Sequence logos derived from RNA SNVs in muscles of the BE3 and YE1-BE3-FNLS mice. Analysis was performed on generated RNA-seq data using cDNA, and thus every T depicted should be considered a U in RNA. **g** Kyoto Encyclopedia of Genes and Genomes (KEGG) pathway enrichment analysis of the RNA SNVs detected in muscles of the BE3 transgenic mice. Genes with a change in expression > 2.0-fold compared with the baseline value and with a *P* value < 0.05 were selected for analysis. Source data are provided as a Source Data file.

We also assessed the off-target effects of BE3, YE1-BE3-FNLS, and ABE7.10^F148A^ based on RNA-seq of the transcriptomes of eight tissues from 15 transgenic and WT mice. Compared with the GFP mice, all eight examined tissues of the BE3 transgenic mice showed higher numbers of RNA SNVs, and five of which showed significant differences (*P* < 0.05; Supplementary Fig. 5a). Among the eight tissues, the highest number of RNA SNVs were found in muscles (Fig. 3a). In addition, we found a positive correlation (*R*^*2*^ = 0.4) between the RNA SNV numbers and the expression level of BE3 across various tissues in the transgenic mice (Fig. 3b). Moreover, genes with higher expression levels showed higher numbers of RNV SNVs in muscles of the BE3 mice (Fig. 3c), suggesting that highly expressed genes are prone to harbor RNA SNVs. YE1-BE3-FNLS muscles also had more RNA SNVs compared to GFP muscles, while the number of RNA mutations in muscles of the ABE7.10^F148A^ transgenic mice were similar to the GFP mice (Fig. 3a and Supplementary Fig. 5a).

An average of 51% and 34% RNA SNVs detected in the BE3 and YE1-BE3-FNLS transgenic mice were shared by more than one sample in the group, respectively (Fig. 3d), and these common SNVs were significantly enriched in highly expressed genes (Supplementary Fig. 5b). Moreover, 88% and 68% of the RNA SNVs identified in the muscles of BE3 and YE1-BE3-FNLS transgenic mice were mutated from C-to-U or G-to-A, which was not observed in the GFP (24%) or the ABE7.10^F148A^ (27%) group (Fig. 3e). The C-to-U and G-to-A mutation preference was also observed in the heart showing high expression of BE3 (Supplementary Fig. 5c and Fig. 1e). Consistently, we also found a motif WCW (W = A or T) enrichment of RNA SNVs in the muscles of BE3 and YE1-BE3-FNLS transgenic mice (Fig. 3f). Sanger sequencing verified the presence of C to U or G to A mutations on the RNA but not DNA level (Supplementary Fig. 6 and Supplementary Table 5).

Given the extensive off-target edits in RNA level, we then examined the adverse effect of these off-target SNVs by KEGG analysis. The results showed that genes with RNA SNVs in muscles of the BE3 mice revealed enrichment for insulin signaling, lipid metabolism and cancer related pathways (Fig. 3g). Taken together, these results indicate that BE3 and YE1-BE3-FNLS induced RNA SNVs in vivo, and the number of RNA SNVs is positively correlated with the expression levels of the base editor.

### Abnormal phenotypes were observed in transgenic mice with CBE overexpression

**Fig. 4:**
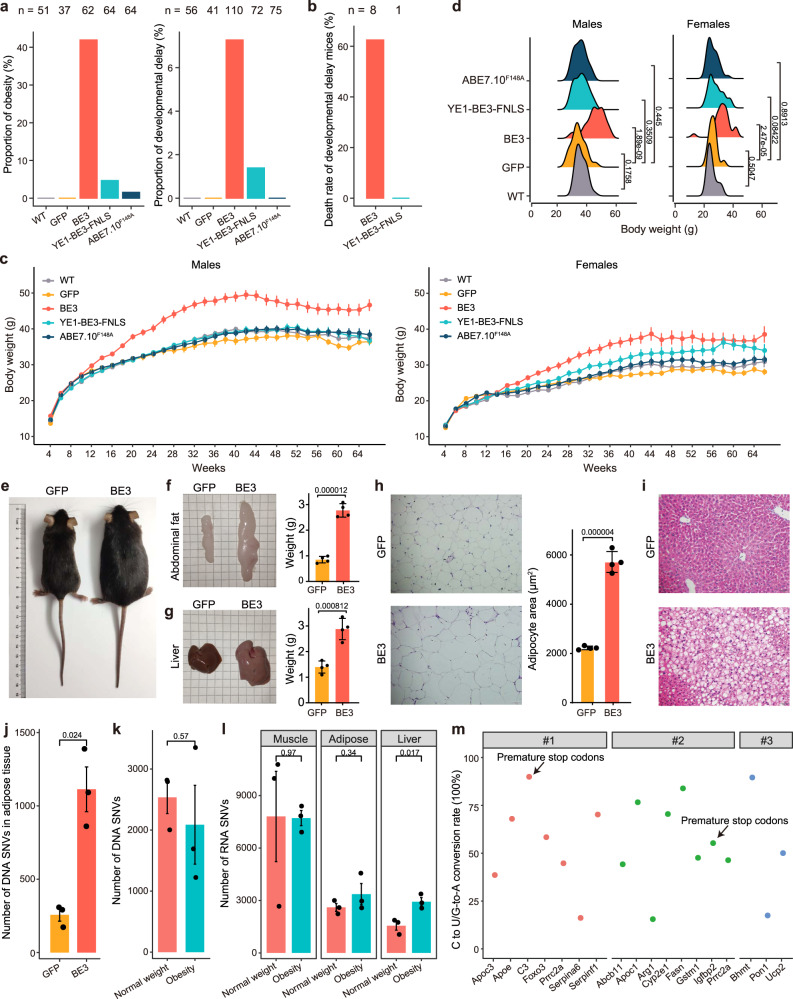
The phenotypic effects of BE3, YE1-BE3-FNLS and ABE7.10^F148A^ in the transgenic mice. **a** The proportions of mice with obesity at 30-week-old or developmental delay at 8-week-old in WT, BE3, YE1-BE3-FNLS and ABE7.10^F148A^ groups. Obesity: the body weight greater than the mean + 3 SEM of WT group; Developmental delay: the body weight lower than the mean − 3 SEM of WT group. **b** Death rate of developmental delay mice before 12 weeks in BE3 and YE1-BE3-FNLS groups. **c** The body weight measurements of the WT (*n* > 13 males and 11 females), GFP (*n* > 12 males and 9 females), BE3 (*n* > 22 males and 16 females), YE1-BE3-FNLS (*n* > 20 males and 12 females) and ABE7.10^F148A^ (*n* > 22 males and 21 females) mice every two weeks from 4 to 66 weeks at age. Data are presented as mean ± SEM. *P* value statistics are shown in Supplementary Fig. 7e and listed in Supplementary Data. 1. **d** The distribution of body weights of male and female mice in five groups at 30-week-old. **e** Representative photos of 30-week-old male mice in GFP and BE3 groups. **f**, **g** Representative photos and weights of abdominal fat and liver tissues in GFP and BE3 mice (30-week-old, male). **h** H&E staining of abdominal fat, and morphometric analysis of the area of abdominal adipocytes in GFP and BE3 mice. **f**–**h**, Data are presented as mean ± SEM (*n* = 4). *P* values were calculated by two-sided unpaired *t*-test. **i** H&E staining of liver tissues in GFP and BE3 mice. **j** Number of DNA SNVs in abdominal fat tissues from GFP and BE3 groups. **k** Number of DNA SNVs in BE3 obesity and normal weight mice. **l** Number of RNA SNVs in muscle, adipose, liver from BE3 obesity and normal weight mice. **j**–**l** Data are presented as mean ± SEM (*n* = 3). *P* values were calculated by two-sided unpaired *t*-test. **m** Premature stop codons and non-synonymous of genes induced by off-target C-to-T editing in obesity mice that are implicated in obesity. Source data are provided as a Source Data file.

Considering the substantial off-target DNA and RNA effects of base editors, we next monitored the phenotypes of ~400 transgenic mice over 15 months to explore the potential side effects of long-term BE expression. We firstly analyzed the fertility of the transgenic mice and found no difference in the total numbers of litters, pups per litter, gender ratio and birth weights between BE and GFP transgenic mice (Supplementary Fig. 7a–d). However, we found a significantly higher proportion of BE3 transgenic mice showed abnormal phenotypes including obesity and developmental delay (Fig. 4a). Notably, most of these mice with developmental delay lived no longer than 12 weeks (Fig. 4b). The monitoring on body weight over 66 weeks revealed that BE3 transgenic mice tended to be significantly heavier than GFP group starting from 16 weeks in both sexes (Fig. 4c and Supplementary Fig. 7e–f). Specifically, the body weights of BE3 transgenic mice reached 45.6 ± 6.5 (mean ± s.e.m.) grams (g) in male and 32.7 ± 5.7 g in females at 30 weeks, significantly heavier than those in the GFP mice (34.4 ± 4.7 g in males and 26.0 ± 2.8 g in females) (Fig. 4d). The YE1-BE3-FNLS female mice also showed evidently higher weight gain than the GFP female mice after 30 weeks (Fig. 4c and Supplementary Fig. 7f). The average weights of ABE7.10^F148A^ transgenic mice showed no difference compared with GFP mice in either sex (Fig. 4c and Supplementary Fig. 7e–f).

To further characterize the roles of BE3 expression in transgenic mice with obesity, we collected inguinal adipose and liver tissues from 30-week-old BE3 and GFP male mice (Fig. 4e). Compared with GFP group, BE3 transgenic mice showed larger size (2.8 g vs 0.9 g) and significantly heavier weight (2.9 g vs 1.4 g) of the inguinal adipose and liver tissues (Fig. 4f, g). Histopathological analysis showed that the adipocyte sizes were significantly larger in the adipose tissues of BE3 transgenic mice (Fig. 4h). Besides, fatty deposits and hepatic dysplasia were observed in livers of BE3 mice by hematoxylin and eosin (H&E) staining (Fig. 4i). We then performed RNA-seq on adipose tissues from the BE3 and GFP transgenic mice, and principal component analysis (PCA) revealed a clear distinction of transcriptome profiles between BE3 and GFP transgenic mice (Supplementary Fig. 8a). Further differential expression analysis identified 2765 upregulated and 1647 downregulated genes in the BE3 transgenic mice (Supplementary Fig. 8b). The downregulated genes were enriched in fatty acid and lipid metabolic pathways (Supplementary Fig. 8c).

To explore the underlying mechanism of the obesity phenotypes, we next performed WGS on adipose tissues from the BE3 and GFP transgenic mice. A significantly higher number of DNA mutations was identified on the genome of BE3 in comparison with the GFP transgenic mice (Fig. 4j). None of these mutations were located in the coding sequence of monogenic obesity genes^25^, but they were significantly enriched in the metabolic processes, insulin secretion, lipid localization and storage pathways (Supplementary Fig. 9a). In addition, we randomly selected three BE3 transgenic mice showing obesity (60.3 g on average) and three with normal weight phenotype (34.3 g on average) (Supplementary Fig. 9b), we firstly analyzed the targeted deep sequencing data to identify the integration sites and copy numbers of BE3 in the genome. The results showed that the average copy numbers in obesity were similar between obesity and normal weight groups (4.7 vs. 5.0; *P* = 0.77) (Supplementary Fig. 9c). In addition, BE3 was integrated into the intergenic or intronic regions of the mouse genome in both overweight and normal weight mice, and all the integration sites in overweight group were overlapped with those with normal weight (Supplementary Table 6 and Supplementary Fig. 9d). These results suggested that the abnormal phenotype was not associated with the copy numbers or integration sites of BE3 in mice. We next compared the number of DNA SNVs from WGS data between the obesity and normal weight mice and found no significant difference (Fig. 4k). Then we analyzed the RNA-seq data, the mRNA expression analysis of BE3 showed no significant difference between two groups in muscle, adipose and liver tissues (Supplementary Fig. 9e). Notably, the numbers of RNA SNVs in the liver of mice with obesity were significantly higher than those with normal weight (Fig. 4l). Furthermore, we performed GO and KEGG pathway enrichment analysis of genes containing the RNA SNVs uniquely identified in the liver of obesity mice, and found these genes were significantly enriched in fatty acid metabolic process, lipid catabolic process, and carbon metabolism pathways (Supplementary Fig. 9f, j). Specifically, we found off-target C-to-T editing in obesity mice generated premature stop codons and non-synonymous mutations on obesity and metabolic disorder-associated genes, e.g., *Igfbp2*, *C3*, *Apoe*, and *Apoc3* (Fig. 4m). Obesity has been reported as a complex trait caused by multiple genomic sites^25,26^, our results suggested that a higher number of BE3 induced off-target RNA mutations on obesity-associated genes might be responsible for the obesity phenotype in mice with BE3 overexpression.

We also found that two dead mice with developmental delay in the BE3 group carried 5 times higher number of DNA mutations than that of the GFP mice (Fig. 4b and Supplementary Fig. 10a), and these mutations located genes were significantly enriched in the nervous system, cell and tissue development-related pathways (Supplementary Fig. 10b). Taken together, the abnormal phenotypes including obesity and developmental delay observed in transgenic mice might possibly be caused by the accumulation of BE3 induced off-target mutations.

## Discussion

In gene therapy, using the leading AAV delivery platform, BEs could maintain in cells for 6–12 months or even for many years in vivo^27^. Therefore, long-term and comprehensive evaluation for off-target effects of BEs is necessary for their clinical translation. Here we established a SAFETI method to evaluate the comprehensive and long-term effects of base editors on diverse tissues in vivo by generating transgenic mice. The SAFETI system has several advances in comparison with the previous off-target detection methods. Firstly, it could evaluate the genome-wide and transcriptome-wide off-target mutations in diverse tissues with high sensitivity. In fact, the high-fidelity version of CBE, YE1-BE3-FNLS, was also found to induce numerous RNA mutations using the SAFETI system. Secondly, the SAFETI method could evaluate the phenotypic influence of base editors in vivo. We observed abnormal phenotypes including overweight and developmental delay in mice with permanent CBE overexpression. Thirdly, this system enabled us to monitor the long-term effects of BE expression in vivo. We found RNA SNVs located in tumor-associated genes, which was consistent with the results in HEK293T. While, we observed no tumor occurrence in the transgenic mice during the 15 months monitoring, suggesting the off-target RNA SNVs are not necessarily for the tumor development in vivo. This phenomenon also indicates the importance to detect the off-target effects for safety evaluation in vivo, and longer time monitoring might better answer the relationship between BE expression and aging-related diseases, e.g. cancer. In summary, our SAFETI system is a universal method to systematically evaluate the off-target effects of gene editing tools in vivo, including the newly developed optimized base editors ABE8e^28^, ABE8e-N108Q/L145T^29^, programmable C-to-G base editors^30–33^, and prime editors^34,35^.

Using the SAFETI system, we found that permanent genomic BE3 overexpression induced off-target SNVs with adverse phenotypic effects in transgenic mice, which was an overlooked aspect of the potential side effects of base editors. The obesity and developmental delay phenotypes were possibly be caused by the accumulation of off-target mutations induced by base editing in vivo. These off-target mutations were randomly generated and might occur in many genes, the interaction of which might induce complex diseases^36,37^. Nevertheless, the adverse phenotypic effect might be induced by permanent BE3 overexpression in all cells of the body from early development onwards. This raised the concern of long-term expression of base editors in the therapeutic applications, so we next delivered the base editors into mice with clinically relevant AAV delivery method. We examined the transgene copy numbers, transgene expression and off-target RNA SNVs in mice with YE1-BE3-FNLS delivered by AAV, and found lower copy numbers and expression levels of the transgene as well as lower numbers of off-target RNA SNVs as compared to those in YE1-BE3-FNLS transgenic mice (Supplementary Fig. 11). In combination with our results showing that the number of off-target mutations was positively correlated with BE3 expression level, somatic therapies where base editors are expressed in a tissue-specific manner at lower doses may not lead to abnormal phenotypes.

It is noteworthy that the expression of YE1-BE3-FNLS was much higher than BE3, but YE1-BE3-FNLS induced much fewer DNA and RNA mutations and no severe phenotypic effects. Similarly, the RNA SNV numbers in ABE7.10^F148A^ mouse line were similar to those in GFP group. These results demonstrated that the high-fidelity versions of both CBE (YE1 mutation in rAPOBEC1) and ABE (F148A mutation in TadA) could significantly reduce the off-target RNA SNVs in vivo as well as in vitro. With the rapid development of newly engineered CBEs or ABEs with low off-target deamination, further studies are needed for evaluating their specificity in vivo.

In this work, we develop SAFETI method for systematically evaluating the off-target effects of gene editing tools in vivo, and our results point directions to lower off-target effects of gene editing tools in gene therapy by future protein engineering for high-fidelity deaminases or by transient delivery methods.

## Methods

### Ethics statement

The study protocol of mouse care and experiments was approved by the guideline of the Life Sciences Ethics Committee of Agricultural Genomics Institute at Shenzhen, Chinese Academy of Agricultural Sciences. All animal studies complied with relevant ethical regulations for animal testing and research.

### Generation of the transgenic mice

We generated seven mouse lines expressing GFP, BE3, YE1-BE3-FNLS, hA3A-BE3, ABE7.10, ABE7.10^F148A^, or *Tyr* sgRNA using *piggyBac* transposon integration system in the zygotes of C57BL/6J strain using procedures previously described^14^. The transposon vectors for GFP and base editors consist of a CAG promoter followed by one of the base editors linked via P2A peptide to an enhanced green fluorescent protein (eGFP). The *Tyr* sgRNA transposon vector included mCherry followed by U6-*Tyr* sgRNA cassette. The transposon vectors were injected into C57BL/6J zygotes together with PBase mRNA. Then the zygotes were cultured in KSOM medium at 37 °C under 5% CO_2_ in air for 24 h, and then transferred into oviducts of the pseudopregnant female ICR mice. We verified the successful integration of transgenes by fluorescence detection and PCR genotyping with DNA purified from tails of the offspring (Primers are detailed in Supplementary Table 1). Then the mice with successful integration of the corresponding transgenes were intercrossed to establish the germline-transmitted founders. Cross-mating of founders resulted in transgenic mouse lines expressing GFP or one of the base editors.

### Analysis of transposon integration sites and copy numbers by HTS sequencing and PCR

18 samples of tails were taken from 8-week-old GFP, BE3, YE1-BE3-FNLS and ABE7.10^F148A^ mice, 30-week-old normal and obese BE3 mouse for gDNA extraction. Integration sites were recovered from gDNA by shearing DNA with sonication, ligating DNA linkers to the broken DNA ends, then carrying out nested PCR amplification using sample-specific primers (Supplementary Data. 2) that bound to the ligated adapter and the 5-arm/3-arm sequences in *PB* transposon vectors, as previously reported^38^. PCR products were purified using universal DNA purification kit (TIANGEN) for HTS sequencing. The reads were mapped to the sequence of 5-arm or 3-arm, and the unmapped side sequence of the splited reads were mapped to mouse genome. The integration sites were predicted from mapping position. The predicted integration sites at 5 arm or 3 arm terminals were separately confirmed by integration site-specific primers (Supplementary Data. 3). Then integration site-specific forward and reverse primers which binding genome sequence were used to identify single or biallelic integration (Supplementary Data 3). The total number of single (considered as one copy) and biallelic (considered as two copies) integration sites was calculated as copy number in each sample.

### RNA isolation and RNA-seq

Total RNA of each sample was extracted using Trizol (Ambion) according to the standard protocol. The mRNAs were fragmented and converted to cDNA using random hexamers or oligo(dT) primers. The 5′ and 3′ ends of cDNA were ligated with adaptors, and correctly ligated cDNA fragments were enriched and amplified by PCR^4^. High-throughput mRNA sequencing was carried out using Illumina Novaseq 6000 and ~22 million reads were produced for each library.

FastQC (v0.11.8) and Trimmomatic (v0.39)^39^ were used for quality control. Qualified reads were mapped to the mouse reference genome (mm10) by STAR (v2.1.7)^40^ with two pass model. The BAM files were then sorted and PCR duplicates were marked using Picard (v2.5.7.0). The variants were called by GATK (v4.1.5). The variants with depth <20, quality score <30, QD < 2 and FS > 30 were filtered. We filtered variants detected in the same organ of WT mice, and those in the dbSNP142 dataset or located in highly complex regions from the following analysis. We also filtered out variants found in the corresponding DNA variants to rule out the influence of genetic background if we performed WGS on the same sample. To make functional annotations for the identified variants, we applied annovar (v2020-06-08) to annotate the detected SNVs and indels using RefSeq database of mm10. The adjacent 3-bp sequences of the off-target RNA SNVs were extracted from the reference genome and subjected to motif prediction using ggseqlogo^41^. HTseq (v0.11.3) and GenomicFeatures were used to estimate the gene-expression levels on the alignment file with default parameters and gene abundances were reported in FPKM (Fragments Per Kilobase of exon model per Million mapped fragments)^42^.

The differential expression analysis was conducted using DESeq2^43^, and differentially expressed genes were defined using the criteria: absolute log2 transformed fold-change > 1 and *P* value < 0.05. The GO and KEGG enrichment analysis were performed using clusterProfiler^44^.

### Protein isolation and western blot

Proteins were isolated from the examined tissues using radioimmunoprecipitation assay lysis buffer (P0013C; Beyotime) and quantified using a BCA assay (P0012; Beyotime). Equal amounts of proteins (50–100 µg) were electrophoresed on SDS‐PAGE and transferred to PVDF membranes (ISFQ00010; Merck-Millipore), blocked with 5% nonfat milk for at least 1 h at room temperature, and reacted with primary antibodies including Cas9 (7A9-3A3) mouse mAb (14697S, Cell Signaling Technology, 1:1000) and HRP-conjugated Beta Actin (2D4H5) monoclonal antibody (HRP-66009, Proteintech, 1:5000) overnight at 4 °C. The PVDF membrane was then washed in Tris-buffered saline containing 0.1% Tween-20 (TBST) and incubated for 1 h with horseradish peroxidase-conjugated goat anti-mouse IgG (SA00001-1, Proteintech, 1:5000). After washing with TBST, the membrane was treated with the Pierce™ ECL western blot analysis substrate (32109; Thermo Fisher Scientific).

### Histological staining

Tissues were fixed in 4% PFA overnight, washed with PBS, dehydrated in a gradient series of alcohol solutions, and embedded in paraffin. These paraffin-embedded tissues were then sectioned (5 μm) before being deparaffinized and rehydrated. Tissue sections were stained with hematoxylin, incubated in bluing solution, counterstained with eosin, dehydrated, and equilibrated with xylene. Glass coverslips were mounted with Permount Mounting Media (SP15-100; Thermo Fisher Scientific). Sections were photographed under bright-field microscope photograph system (Leica Microsystems).

### The AAVs cloning, production, and in vivo injection

The AAV vectors include U6-*Hpd* sgRNA vector, U6-*Dmd* sgRNA vector, N-YE1-BE3-FNLS vector, and C-YE1-BE3-FNLS vector between AAV serotype 2 ITRs. U6-*Hpd* sgRNA and U6-*Dmd* sgRNA vector contain the U6 promoter for *Hpd* or *Dmd* sgRNA transcription, CAG promoter, tdTomato, WPRE and SV40 poly(A) sequence. N-YE1-BE3-FNLS vector contain U6 promoter for *Hpd* sgRNA transcription, EFS promoter, YE1-rApobec1, N-terminal nCas9 (573 sites), N-intein and SV40 poly(A). C-YE1-BE3-FNLS vector contain C-intein, C-terminal nCas9 (574 sites), P2A, eGFP and bGH poly(A). The AAV vectors together with helper and pAAV8 or pAAV9 plasmids were transfected into HEK293T cells using polyethyleneimine (PEI). Viral particles from the media and cells were harvested, purified, and concentrated 3–5 d after transfection. AAV titers were determined by quantitative real-time PCR assays.

The 8-week-old CBE transgenic mice were injected intravenously with 5 × 10^11^ viral genomes (vg) of AAV8: U6-*Hpd* sgRNA per mouse via tail vein. The 8-week-old C57BL/6J mice were injected intravenously with 5 × 10^11^ vg of AAV8:N-YE1-BE3-FNLS and 5 × 10^11^ vg AAV8:C-YE1-BE3-FNLS per mouse via tail vein. CBE transgenic mice were sacrificed two weeks post-injection of AAV8:U6-*Hpd* sgRNA, and primary hepatocytes were isolated by standard two-step collagenase perfusion method purified by 40% Percoll (Sigma) at low-speed centrifugation (1000 rpm, 10 min)^45^. The GFP and tdTomato-positive hepatocytes were isolated using FACS (Flow Jo v10.0.7) for PCR analysis. C57BL/6J mice were sacrificed at 1-, 2-, and 3-week post-injection of AAV8:N-YE1-BE3-FNLS and AAV8:C-YE1-BE3-FNLS, and livers were sampled for droplet digital PCR and RNA-seq. The 8-week-old ABE transgenic mice were locally administered with 1 × 10^11^ vg (per leg per mouse) into the tibialis anterior (TA) muscle. TA muscles were collected after two weeks of AAV injection for Sanger and deep-sequencing analyses.

### Amplification and HTS sequencing of genomic DNA samples

Genomic DNA of hepatocytes or target tissues was isolated using the DNeasy Blood and Tissue Kit (Qiagen). The genome sequences of targeted sites were amplified by nested PCR using site-specific primers (Supplementary Table 4). PCR products were purified using universal DNA purification kit (TIANGEN). The amplicons were ligated to adapters and sequenced using Illumina Novaseq 6000. Sequencing data were analyzed according to the method reported before^8^. Briefly, the raw data were demultiplexed by fastq-multx^46^, and the reads for each sample were aligned to the reference target sequence by CRISPResso2 (v2.0.32)^47^. The on-target editing efficiency for each target site was then calculated using in-house scripts.

### WGS on muscle and data analysis

Genomic DNA was isolated from different tissues using the DNeasy Blood and Tissue Kit (Qiagen). WGS was performed at mean coverages of 36× by MGI2000. The raw data were qualified by SOAPnupk (v2.1.6)^48^ with default parameters. Qualified reads were mapped to the mouse reference genome (mm10) by BWA mem (v0.7.12)^49^. The BAM files were then sorted and PCR duplicates were marked using Picard (v2.5.7.0). To identify the genome wide de novo variants with high confidence, we conducted single nucleotide variation calling using three algorithms, Mutect2 (v4.1.5), Lofreq (v2.1.5)^50^, and Strelka (v2.7.1)^51^. Whole genome de novo indels were detected using Mutect2, Scalpel (v0.5.4)^52^, and Strelka in the same way. SNVs and indels supported by all the three algorithms were reserved for the following analysis. For detection of de novo mutations in the offspring, we called variants in each progeny with the parents as normal control.

We filtered variations in the UCSC dbSNP142 dataset or located in high complex regions including UCSC repeat regions from the following analysis. To reduce the germline variations, the variations shared between two individuals expressing the same base editors were also filtered out. To make functional annotations for the identified variants, we applied annovar (v2020-06-08) on both the detected SNVs and indels using RefSeq database of mm10.

The structure variations (SVs) were called by Manta (v1.6.0)^53^, Lumpy (v0.2.13)^54^, and Delly (v0.7.6)^55^ using default parameters. To identify the genome-wide SVs with high confidence, we only reserved those supported by all the three algorithms for the following analysis. To reduce the germline SVs, the SVs shared between two individuals expressing the same base editors were filtered out.

### Phenotypes detection of the transgenic mice

8–10 weeks old of males and females from WT, GFP, BE3, YE1-BE3-FNLS or ABE7.10^F148A^ group were intercrossed. Their offspring identified with successfully integration with the corresponding transgene were monitored and weighed. During the preweaning period before 3 weeks, both males and females were weighed together. After weaning after 3 weeks, male and female mice were put into separate cages for continue observation and body weight measurements every two weeks over 66 weeks. For evaluation of fertility, three breeding pairs of the WT and four transgenic mice at 8–10 weeks old were mated for 6 months. The number of pups per litter, total number of pups and total numbers of both males and females were recorded.

### Droplet digital PCR reactions

For comparison of transgene copy numbers in the transgenic mice and in mice with AAV delivery methods, the transgene copy numbers in mice injected with AAV8:N-YE1-BE3-FNLS and AAV8:C-YE1-BE3-FNLS and in YE1-BE3-FNLS transgenic mice were measured by droplet digital PCR using Bio-rad QX200 ddPCR system (Bio-Rad)^56^. Vector genomes were normalized to mouse Sod2^56^. Sequences of primers and probes are listed in Supplementary Table 7.

### Statistics and reproducibility

Sample sizes are reported in each figure legend. Data are presented as means ± SEM. *P* values were calculated by two-sided, unpaired *t*-test. Differences with a *P* value < 0.05 were considered to be statistically. Statistical details of analyses can be found in the figure legends. No statistical method was used to predetermine the sample size. No data were excluded from the analyses. The experiments were not randomized. The Investigators were not blinded to allocation during experiments and outcome assessment.

### Reporting summary

Further information on research design is available in the Nature Portfolio Reporting Summary linked to this article.

## Supplementary information

Supplementary information

Reporting Summary

Description of Additional Supplementary Files

Supplementary Data 1

Supplementary Data 2

Supplementary Data 3

Peer Review File

## Data Availability

The raw data were deposited in the National Center for Biotechnology Information (NCBI) Sequence Read Archive database under accession code PRJNA831302. All raw sequence data are also available in the China National GeneBank DataBase (CNGBdb) with the accession number CNP0002932. Source data are provided with this paper.

## Source data

Source Data File
