# Supplementary material for: Cytosine base editors induce off-target mutations and adverse phenotypic effects in transgenic mice
Source: Nat Commun. 2023 Mar 30;14:1784. doi: 10.1038/s41467-023-37508-7 (PMC10063651; doi:10.1038/s41467-023-37508-7)
Supplement: Supplementary file 1 — Supplementary information [file 41467_2023_37508_MOESM1_ESM.pdf]

# Supplementary Information

## Cytosine base editors induce off-target mutations and adverse phenotypic effects in transgenic mice

Nana Yan[1,7], Hu Feng[1,7], Yongsen Sun[1,7], Ying Xin[1,2,7], Haihang Zhang[1,7], Hongjiang Lu[1,2], Jitan Zheng[1,3], Chenfei He[1], Zhenrui Zuo[1], Tanglong Yuan[1], Nana Li[1,2], Long Xie[1], Wu Wei[4,5]*, Yidi Sun[6]*, Erwei Zuo[1]*

[1]Shenzhen Branch, Guangdong Laboratory for Lingnan Modern Agriculture, Key Laboratory of Synthetic Biology, Ministry of Agriculture and Rural Affairs, Agricultural Genomics Institute at Shenzhen, Chinese Academy of Agricultural Sciences, Shenzhen, China.
[2]Key Laboratory of Agricultural Animal Genetics, Breeding and Reproduction of Ministry of Education & Key Lab of Swine Genetics and Breeding of Ministry of Agriculture and Rural Affairs, Huazhong Agricultural University, Wuhan China.
[3]State Key Laboratory for Conservation and Utilization of Subtropical Agro-Bioresources, College of Animal Science and Technology, Guangxi University, Nanning, China.
[4]CAS Key Laboratory of Computational Biology, Shanghai Institute of Nutrition and Health, University of Chinese Academy of Sciences, Chinese Academy of Sciences, Shanghai, China.
[5]Lingang Laboratory, Shanghai, China
[6]Institute of Neuroscience, CAS Center for Excellence in Brain Science and Intelligence Technology, Chinese Academy of Sciences, Shanghai, China.
[7]These authors contributed equally: Nana Yan, Hu Feng, Yongsen Sun, Ying Xin, Haihang Zhang

*Corresponding author.

Email: wuwei@lglab.ac.cn (W.W.); ydsun@ion.ac.cn (Y.S.); zuoerwei@caas.cn (E.Z.)

**The PDF includes:**

**Supplementary Figures 1-11**

**Supplementary Tables 1-7**

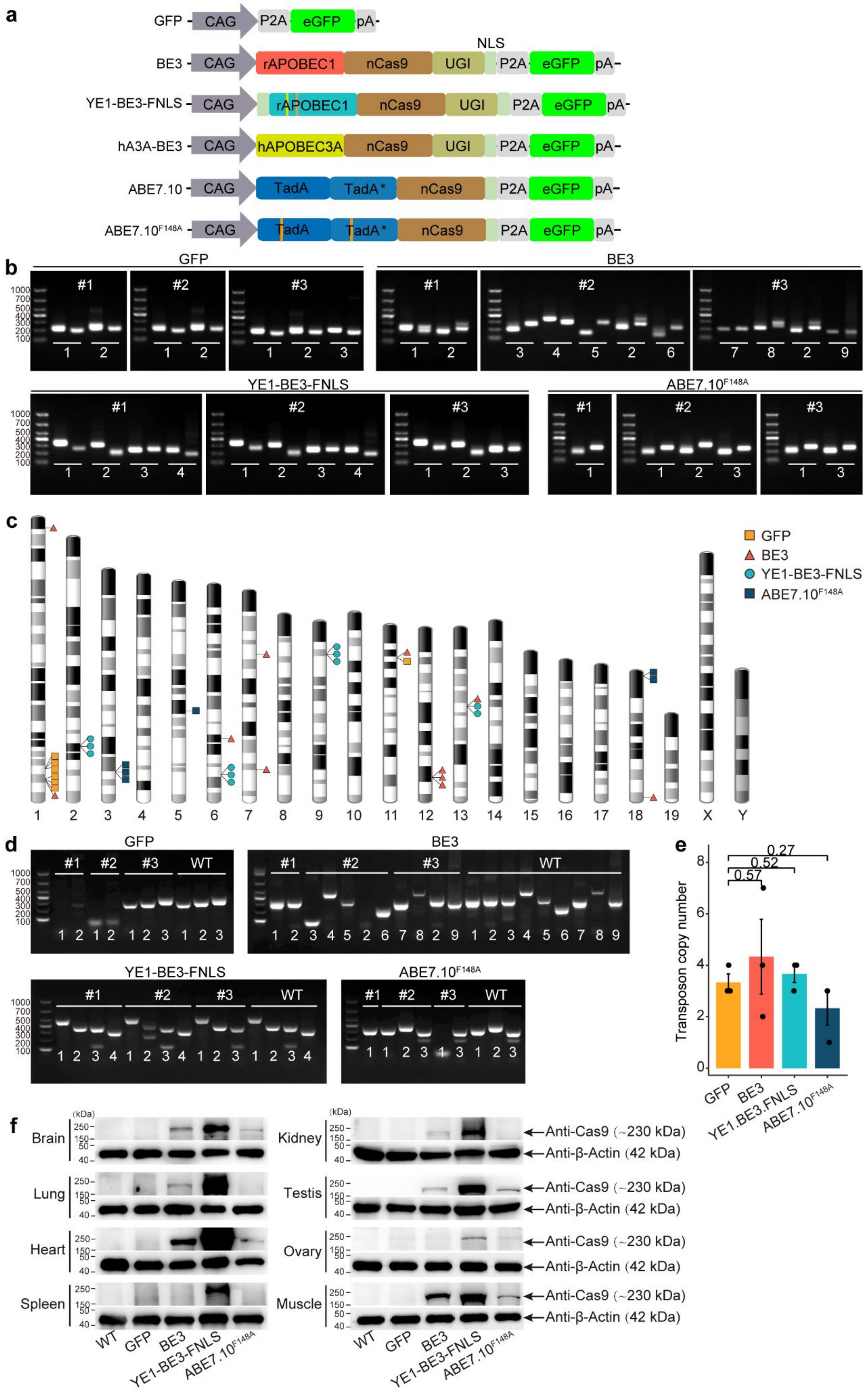

a
GFP
CAG
P2A
eGFP
pA
BE3
rAPOBEC1
nCas9
UGI
NLS
YE1-BE3-FNLS
hA3A-BE3
hAPOBEC3A
ABE7.10
TadA
TadA*
ABE7.10F148A
b
GFP
BE3
YE1-BE3-FNLS
ABE7.10F148A
#1
#2
#3
c
GFP
BE3
YE1-BE3-FNLS
ABE7.10F148A
1 2 3 4 5 6 7 8 9 10 11 12 13 14 15 16 17 18 19 X Y
d
GFP
BE3
YE1-BE3-FNLS
ABE7.10F148A
WT
e
Transposon copy number
0.27
0.52
0.57
GFP
BE3
YE1.BE3.FNLS
ABE7.10F148A
f
(kDa)
Brain
Lung
Heart
Spleen
Kidney
Testis
Ovary
Muscle
Anti-Cas9 (~230 kDa)
Anti-β-Actin (42 kDa)
WT
GFP
BE3
YE1-BE3-FNLS
ABE7.10F148A

**Supplementary Fig. 1 | Generation and identification of GFP, BE3, YE1-BE3-FNLS, ABE7.10$^{F148A}$ transgenic mice. a,** Schematics show the plasmids used for generating transgenic mice. **b,** Electrophoresis of agarose gel to confirm integration sites. The number below electrophoretic bands (left: 5 arm terminal; right: 3 arm terminal) represent corresponding integration sites in three individuals of GFP, BE3, YE1-BE3-FNLS or ABE7.10$^{F148A}$ transgenic mice. **c,** Chromosome location of *PB* insertions in GFP, BE3, YE1-BE3-FNLS and ABE7.10$^{F148A}$ transgenic mice. **d,** Electrophoresis of agarose gel to identify single or biallelic integration in each integration site. **e,** Transposon copy number of GFP, BE3, YE1-BE3-FNLS and ABE7.10$^{F148A}$ transgenic mice. Data are presented as means ± SEM (n = 3). *P* values were calculated by two-sided, unpaired *t*-test. **f,** Western blot analysis showing the protein expression levels of BE3, YE1-BE3-FNLS and ABE7.10$^{F148A}$ in the corresponding transgenic mice compared with WT and GFP mice. Source data are provided as a Source Data file.

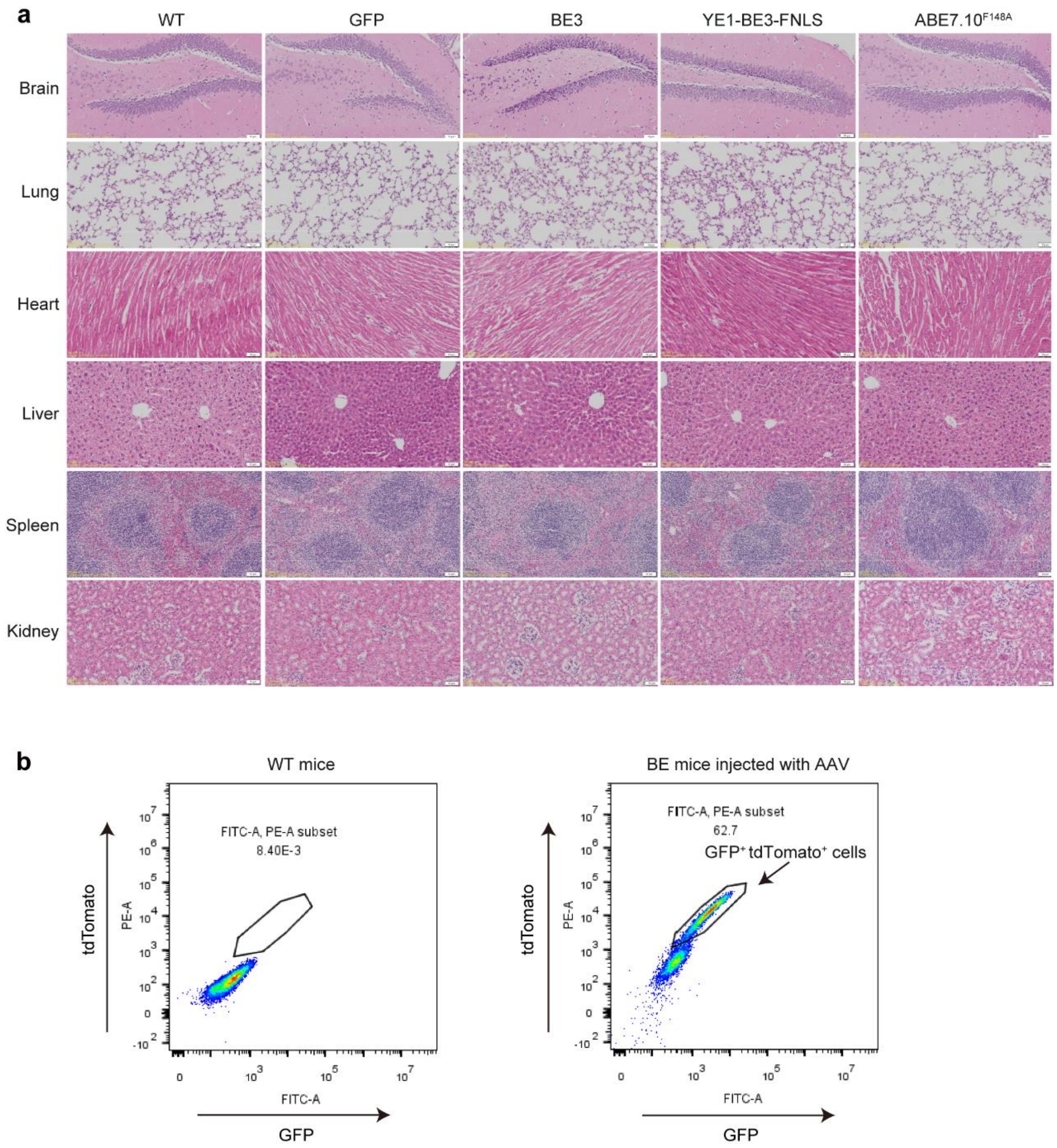


**Supplementary Fig. 2 | H&E staining of the indicated tissues in WT, GFP, BE3, YE1-BE3-FNLS and ABE7.10$^{F148A}$ mice and flow cytometry plots used for sorting GFP$^+$tdTomato$^+$ liver cells. a,** H&E images showed no morphological difference between WT and the transgenic mice. **b,** Representative of the FACS gating strategy in the identification of GFP$^+$ and tdTomato$^+$ cells for on-target editing efficiency evaluation.

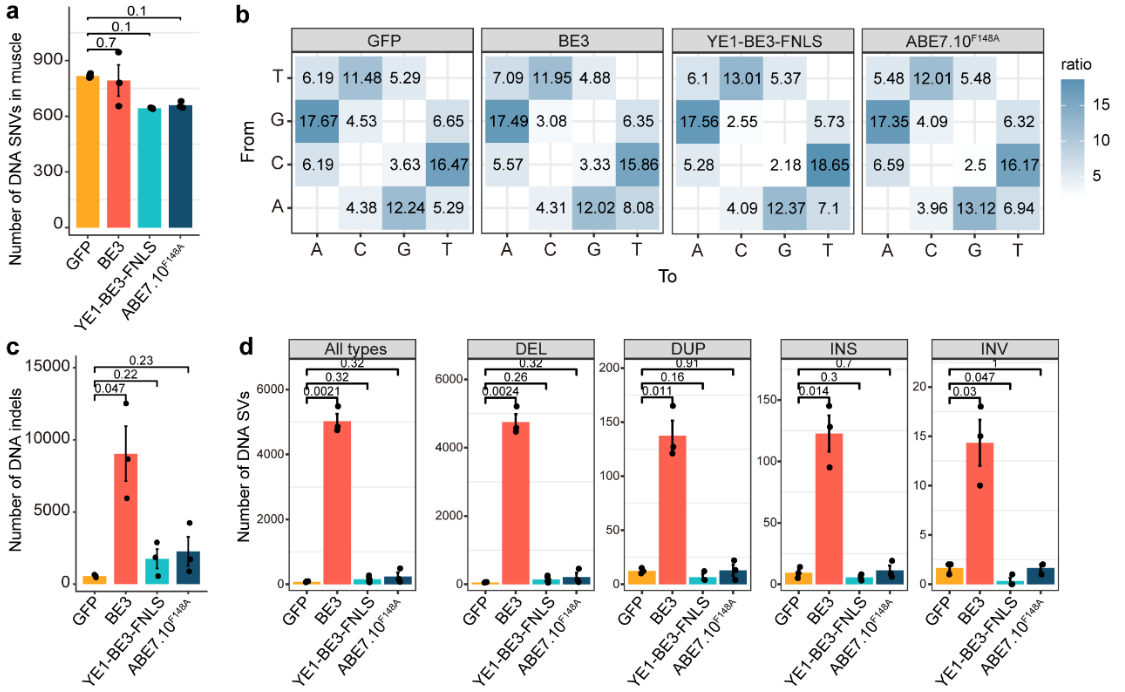


**Supplementary Fig. 3 | WGS analysis in muscles of the GFP, BE3, YE1-BE3-FNLS and ABE7.10$^{F148A}$ transgenic mice. a,** The numbers of DNA SNVs detected in the WT mice with the transgenic mice as control from WGS data. **b,** Distribution of mutation types in muscles of GFP, BE3, YE1-BE3-FNLS and ABE7.10$^{F148A}$ mice. The number indicates the percentage of a certain type of SNVs among all SNVs. **c,** The numbers of DNA indels detected in muscles of GFP, BE3 YE1-BE3-FNLS and ABE7.10$^{F148A}$ mice. **d,** The numbers and distribution of DNA SVs in muscles of GFP, BE3 YE1-BE3-FNLS and ABE7.10$^{F148A}$ mice. Data are presented as mean ± SEM (n = 3). *P* values were calculated by two-sided unpaired *t*-test. Source data are provided as a Source Data file.

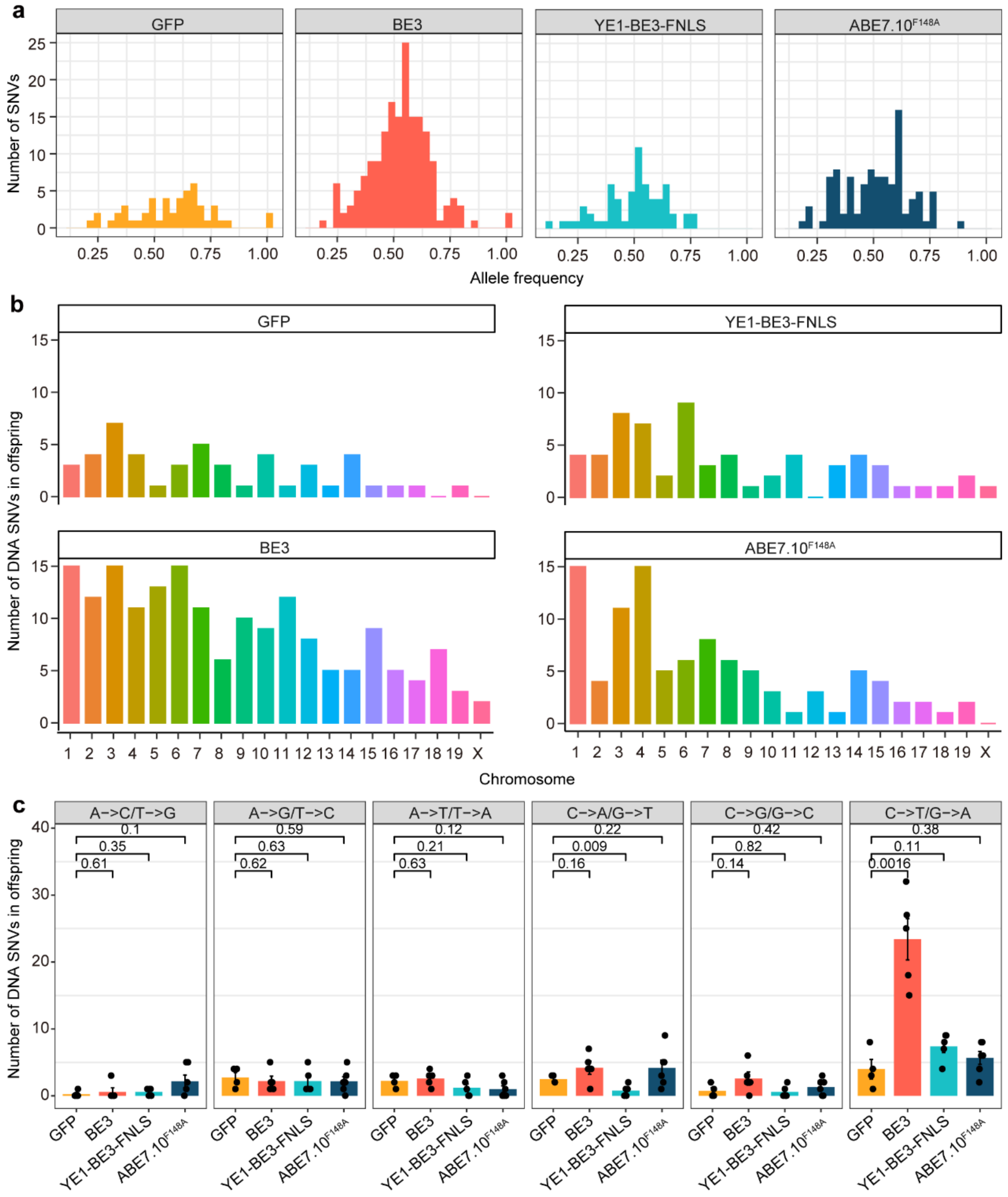


**Supplementary Fig. 4 | WGS analysis for *de novo* off-target SNVs in the offspring of BE transgenic mice. a,** The allele frequency distribution of *de novo* SNVs in the GFP and BE transgenic mice. **b,** Distributions of off-target RNA SNVs on chromosomes of the transgenic mice. **c,** Comparison of *de novo* SNV numbers in the offspring of different transgenic mice in the six indicated mutation types. Data are

presented as mean ± SEM, n = 4, 5, 5 and 6 for GFP, BE3, YE1-BE3-FNLS or ABE7.10$^{F148A}$ group, respectively. *P* values were calculated by two-sided unpaired *t*-test. Source data are provided as a Source Data file.

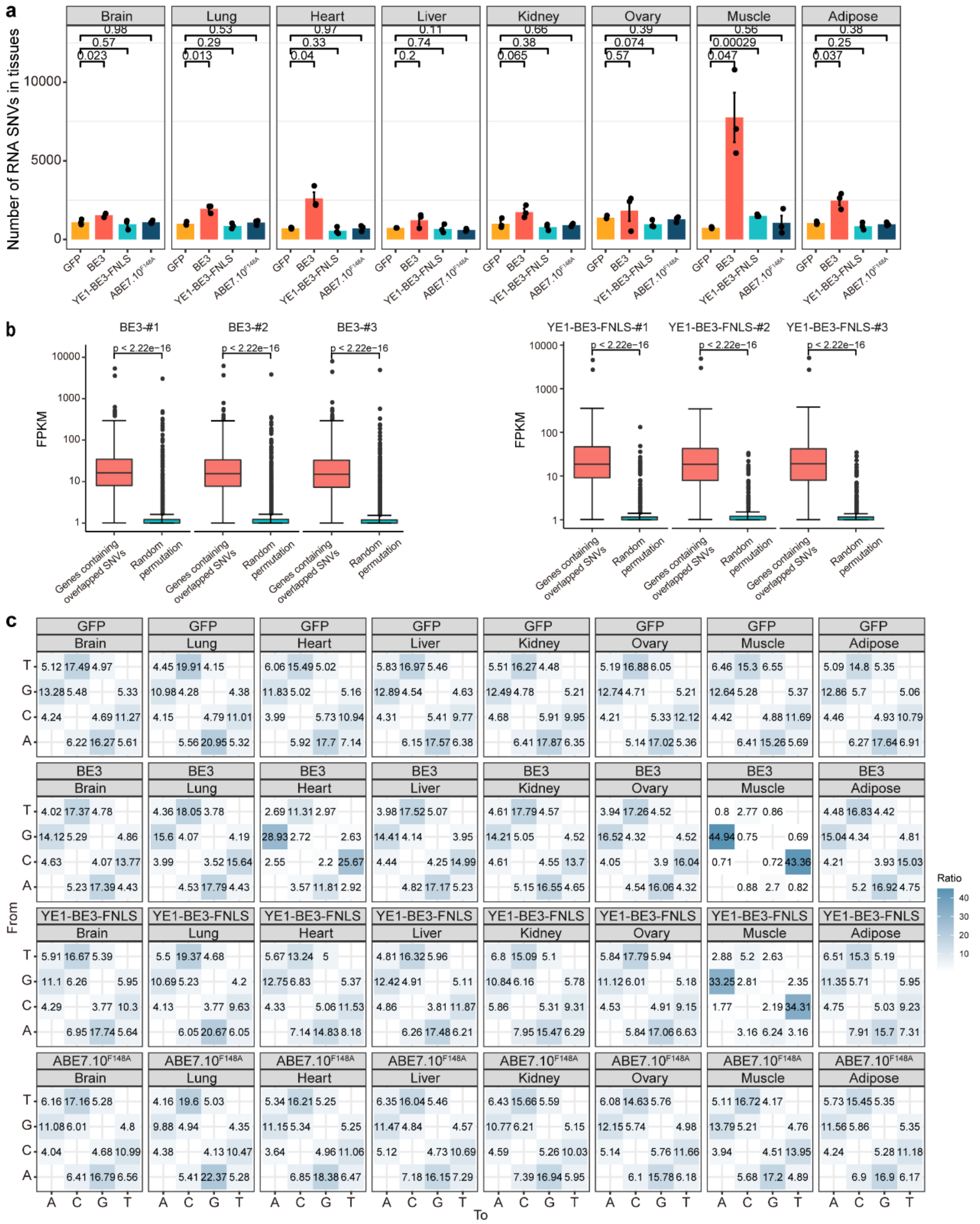


**Supplementary Fig. 5 | RNA SNVs in various tissues of GFP, BE3, YE1-BE3-FNLS and ABE7.10$^{F148A}$ mice. a,** Comparison of RNA SNV numbers in brain, lung, heart, liver, kidney, ovary, muscle, and adipose tissues of the GFP and BE transgenic mice. Data are presented as mean ± SEM (n = 3). *P* values were calculated by two-sided unpaired *t*-test. **b,** Comparison of expression levels for genes containing the

overlapped off-target RNA SNVs and randomly simulated genes in muscles of the BE3 and YE1-BE3-FNLS mice. *P* values were calculated by two-sided unpaired *t*-test. The center line indicates the median, and the bottom and top lines of the box represent the first quartile and third quartile of the values, respectively. Tails extend to the minimum and maximum values. **c,** Distribution of mutation types in the indicated tissues of the GFP, BE3, YE1-BE3-FNLS and ABE7.10$^{F148A}$ mice. The number indicates the percentage of a certain type of SNVs among all SNVs. Source data are provided as a Source Data file.

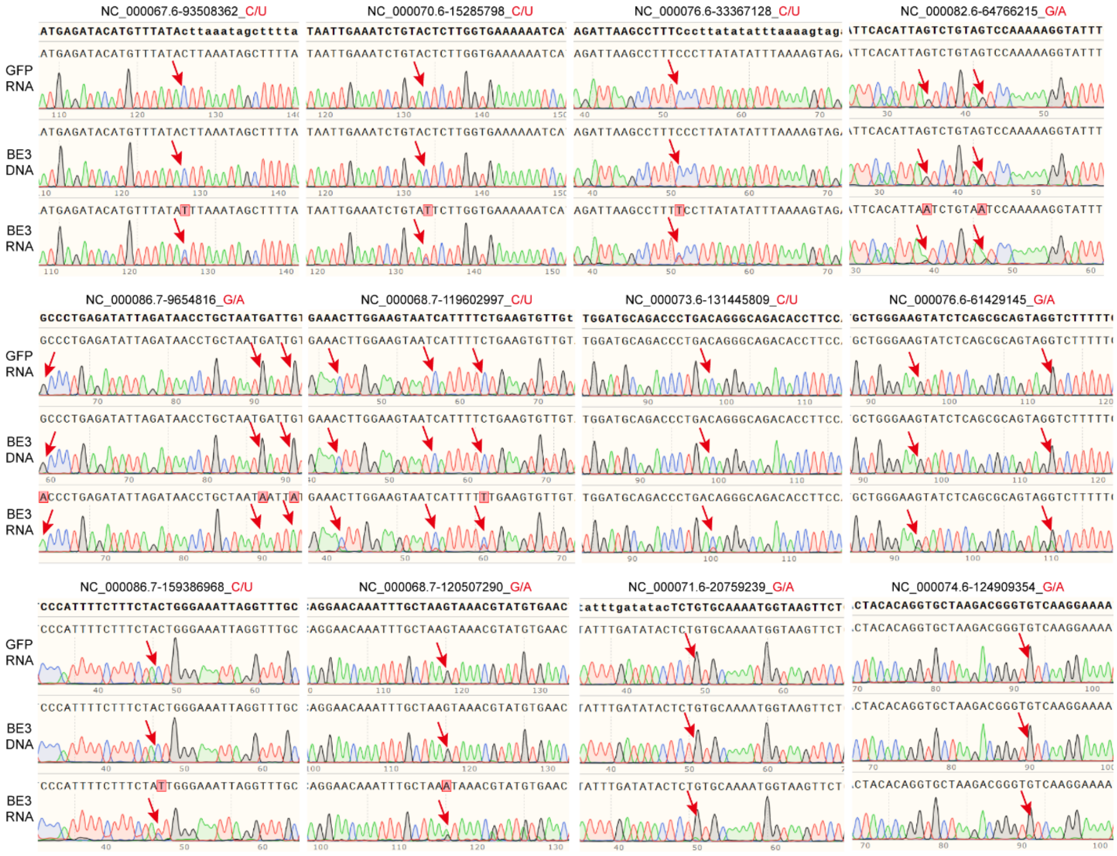


**Supplementary Fig. 6 | Sanger sequencing chromatograms show that the C-to-U RNA SNVs were only observed in RNA but not DNA in the muscles of the GFP and BE3 mice.** Single nucleotide conversions are indicated by red arrows. Primers were listed in Supplementary Table 5.

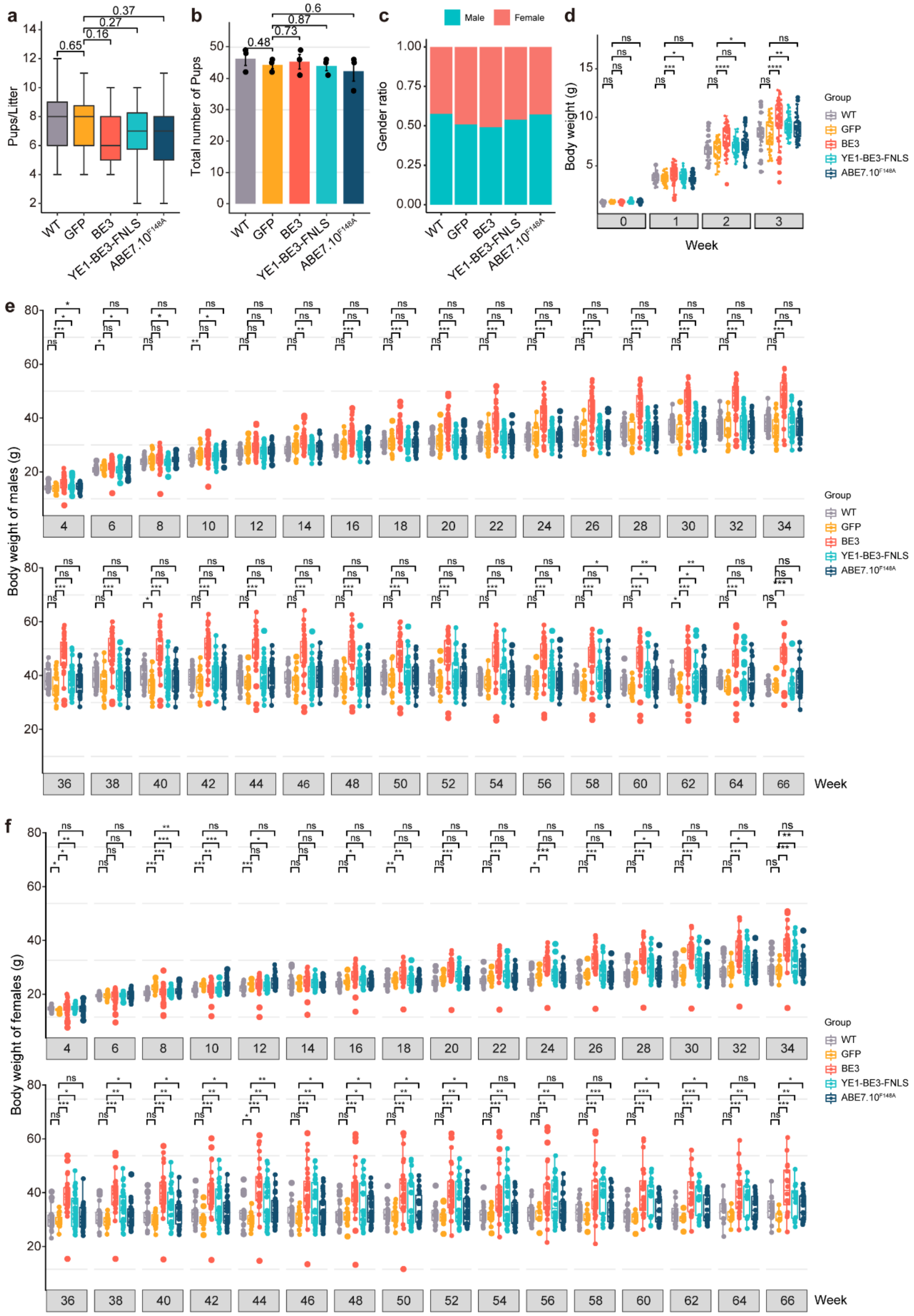


**Supplementary Fig. 7 | The fertility and development phetotype monitoring of transgenic mice. a-c,** Comparison of pups per litter **(a)**, total number of pups **(b)** and

sex ratio **(c)** between GFP and BE transgenic mice. Three breeding pairs of the four transgenic mouse lines were mated for 6 months and the numbers of pups were recorded. Box-and-whisker plots: the center line indicates median, box represents first and third quantiles, and whiskers indicate maximum and minimum values. **d,** Comparison of body weights between WT (n = 46), GFP (n = 48) and BE3 (n = 74), YE1-BE3-FNLS (n = 64) and ABE7.10$^{F148A}$ (n = 63) transgenic mice from birth to 3 weeks. **e, f,** Comparison of body weights between WT (n > 13 for males and > 11 for females), GFP (n > 12 for males and > 9 for females) and BE3 (n > 22 for males and > 16 for females), YE1-BE3-FNLS (n > 20 for males and > 12 for females) and ABE7.10$^{F148A}$ (n > 22 for males and > 21 for females) transgenic male **(e)** and female **(f)** mice from 4 to 66 weeks. Data are presented as mean ± SEM, *$P$ < 0.01, **$P$ < 0.01, ***$P$ < 0.001, ****$P$ < 0.0001 by two-sided unpaired $t$-test. $P$ values are listed in Supplementary Data 1. Source data are provided as a Source Data file.

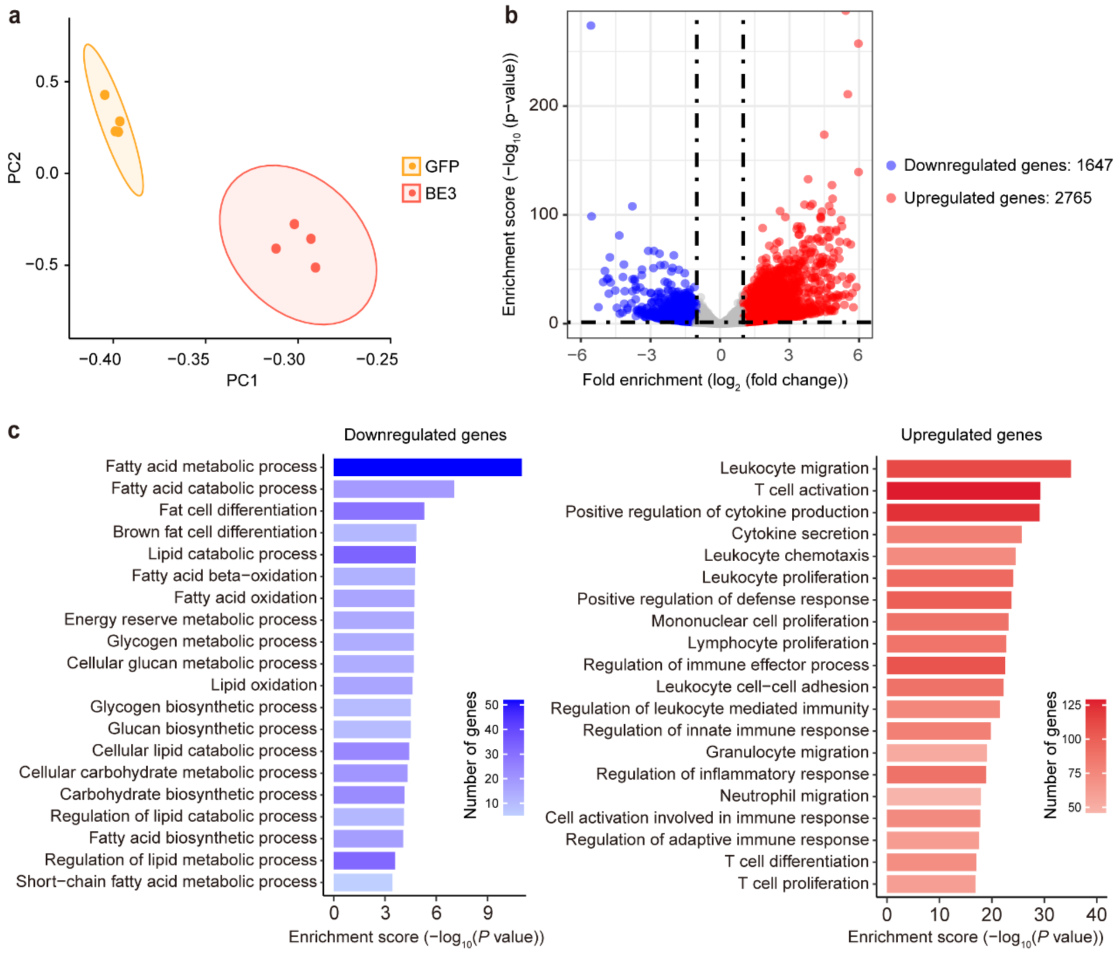


**Supplementary Fig. 8 | RNA-seq analysis for adipose tissues between 30-week-old BE3 and GFP mice. a,** Principal Component Analysis (PCA) analysis of transcriptome-wide gene expression levels between 30-week-old GFP and BE3 male mice. **b,** Volcano plot of the DEGs in adipose tissues between BE3 and GFP mice. **c,** GO analysis of downregulated genes and upregulated genes.

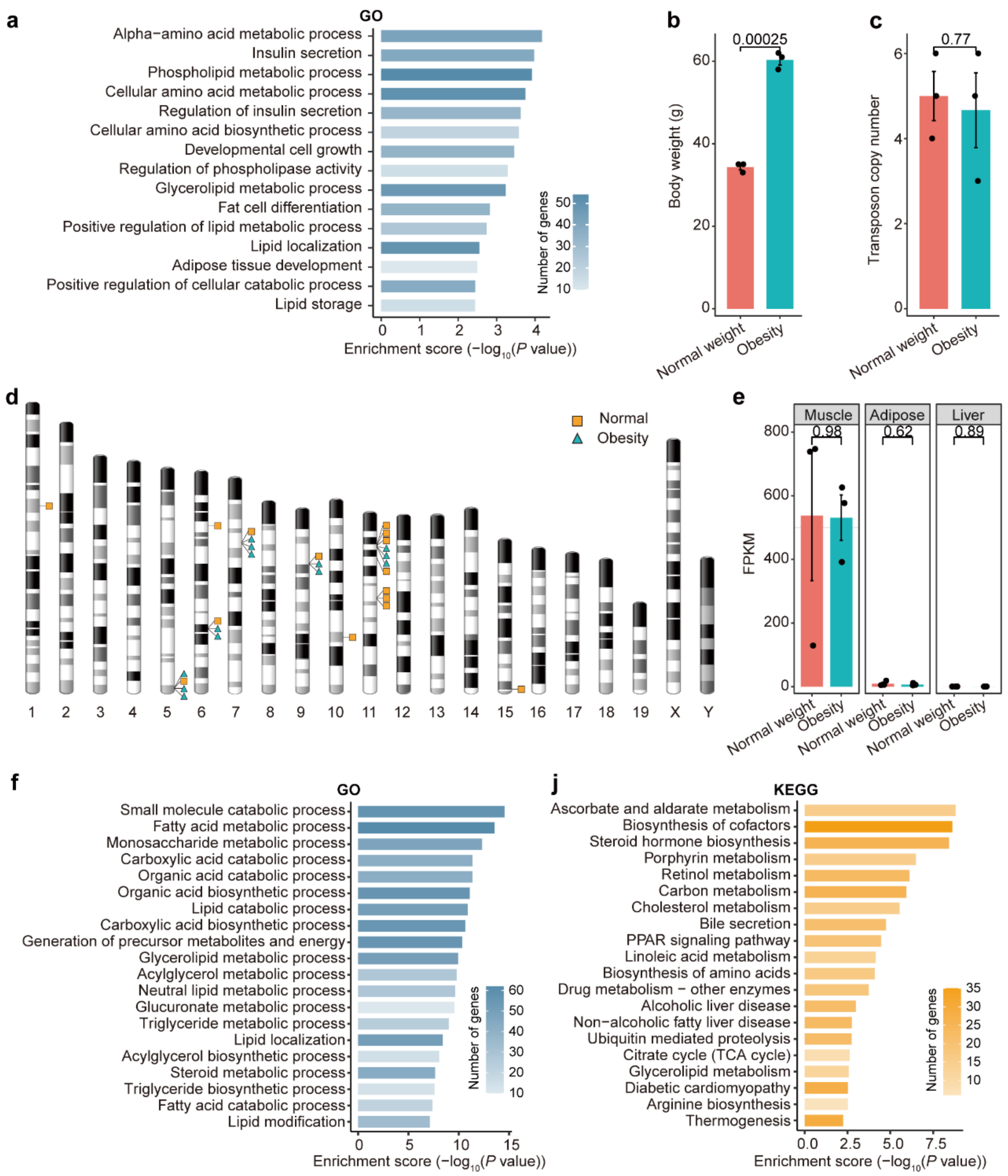


**Supplementary Fig. 9 | Body weight, copy number, integration sites, WGS and RNAseq analysis of BE3 transgenic mice showing obesity and with normal weight phenotype. a,** GO enrichment analysis for DNA SNVs in 30-week-old male mice from BE3 groups. **b,** Body weight of BE3 transgenic mice showing obesity and with normal weight phenotype. **c,** BE3 copy number in obesity and normal weight mice. **d,** Chromosome location of BE3 insertions in obesity and normal weight mice. **e,** The BE3

mRNA expression levels in obesity and normal weight mice. FPKM, Fragments Per Kilobase Million. **f-j,** GO enrichment analysis and KEGG pathway enrichment analysis for unique RNA SNVs detected in liver of obesity mice. Data are presented as mean ± SEM (n = 3). *P* values were calculated by two-sided unpaired *t*-test. Source data are provided as a Source Data file.

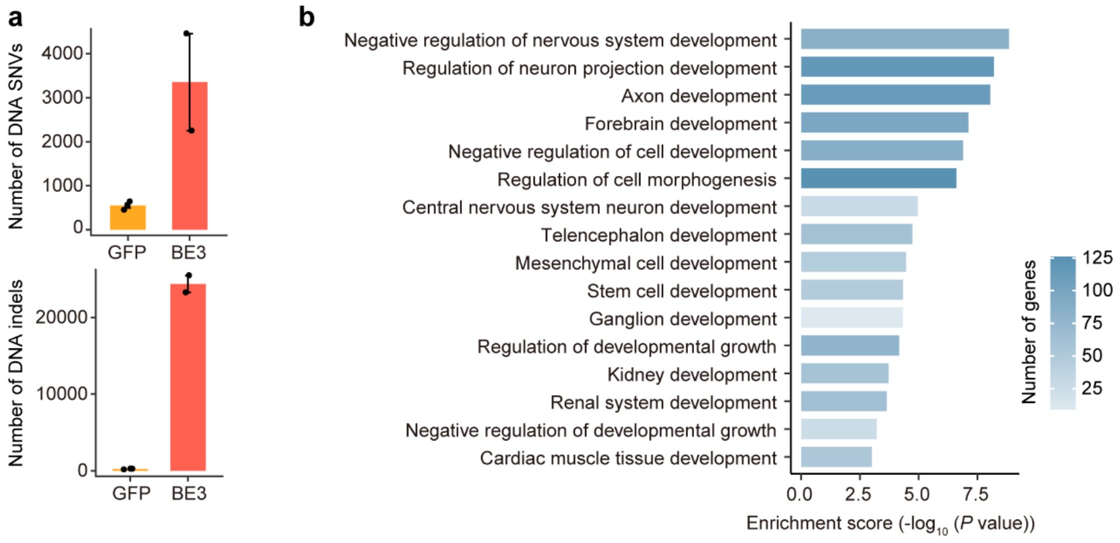


**Supplementary Fig. 10 | WGS analysis of two dead BE3 mice with developmental delay. a,** The numbers of DNA SNVs and indels in the dead BE3 mice (n = 2) compared with the GFP group (n = 3). **b,** GO analysis of genes with DNA mutations in the two dead BE3 mice. Source data are provided as a Source Data file.

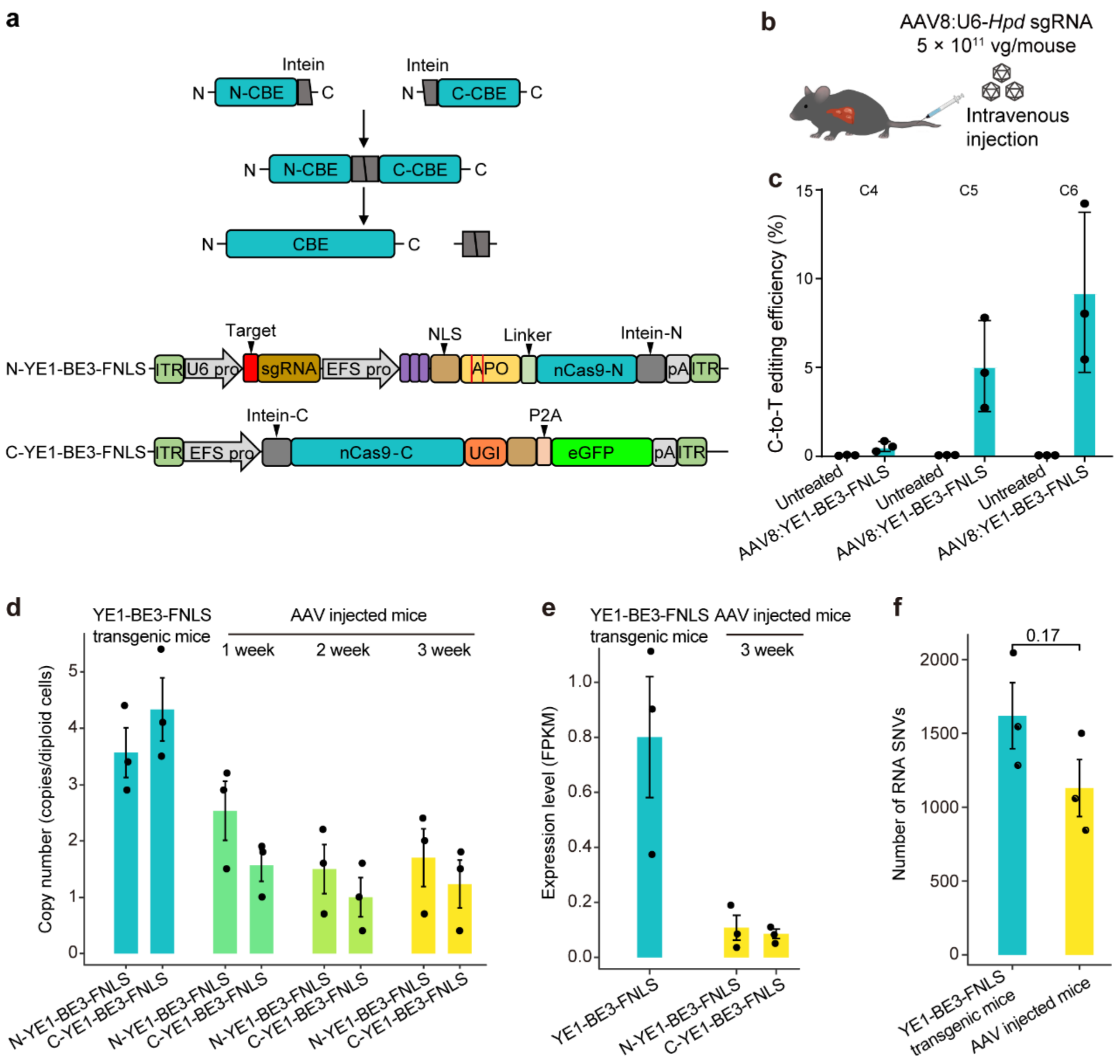


**Supplementary Fig. 11 | Comparison of transgene copy numbers and off-target RNA SNVs in the YE1-BE3-FNLS transgenic mice and in mice with AAV-delivered YE1-BE3-FNLS. a**, Schematic diagram of dual-split YE1-BE3-FNLS for AAV package. **b**, Schematic showing the experimental procedure for delivery of dual-split YE1-BE3-FNLS through AAV8 into mice by tail-vein injection. **c**, Deep sequencing analysis of the C-to-T base editing efficiency at the *Hpd* target site in mice after 3 weeks of AAV injection. **d**, DdPCR detection of YE1-BE3-FNLS copy numbers per diploid cells in the liver of transgenic mice (at 8 weeks old) and AAV-delivered mice (at 1-, 2-, and 3-week post-injection). **e**, Expression levels of transgenes in the

liver of transgenic mice and AAV-delivered mice (at 3-week post-injection). **f**, Numbers of RNA SNVs in the liver of transgenic mice and AAV-delivered mice (at 3-week post-injection). Data are presented as mean ± SEM (n = 3). *P* values were calculated by two-sided unpaired *t*-test. Source data are provided as a Source Data file.

**Supplementary Table 1. Primes for genotyping.**

| Fragment name | Primers | Sequence |
|---|---|---|
| GFP | Forward primer | cctacagatccttaattaagcgacg |
| | Reverse primer | gtagcggctgaagcactgca |
| BE3 | Forward primer | tgctaaccatgttcatgccttc |
| | Reverse primer | taccttctccgagcggttcg |
| YE1-BE3-FNLS | Forward primer | accatgttcatgccttcttc |
| | Reverse primer | cataggttttcagccgttcc |
| hA3A-BE3 | Forward primer | tgctaaccatgttcatgccttc |
| | Reverse primer | taccttctccgagcggttcg |
| ABE7.10 | Forward primer | tgctaaccatgttcatgccttc |
| | Reverse primer | taccttctccgagcggttcg |
| ABE7.10$^{F148A}$ | Forward primer | tgctaaccatgttcatgccttc |
| | Reverse primer | taccttctccgagcggttcg |
| *Tyr* sgRNA | Forward primer | tcaagaactctgtagcaccgcc |
| | Reverse primer | tgacgtcaatggaaagtccc |

**Supplementary Table 2. Insert locations of GFP, BE3, YE1-BE3-FNLS and ABE7.10$^{F148A}$ mice in corresponding transgenic mouse lines.**

| Mouse lines | | Insertion name | Integration site | Location |
|---|---|---|---|---|
| GFP | #1 | GFP-insert 1 | chr1:172468937-172468938 | Gm7804 **Intronic** |
| GFP | #1 | GFP-insert 2 | chr1:179249731-179249732 | Smyd3 **Intronic** |
| GFP | #2 | GFP-insert 1 | chr1:172468937-172468938 | Gm7804 **Intronic** |
| GFP | #2 | GFP-insert 2 | chr1:179249731-179249732 | Smyd3 **Intronic** |
| GFP | #3 | GFP-insert 1 | chr1:172468937-172468938 | Gm7804 **Intronic** |
| GFP | #3 | GFP-insert 2 | chr1:179249731-179249732 | Smyd3 **Intronic** |
| GFP | #3 | GFP-insert 3 | chr11:23139312-23139313 | Gm12059\|1700061J23Rik **Intergenic** |
| BE3 | #1 | BE3-insert 1 | chr11:23139312-23139313 | Gm12059\|1700061J23Rik **Intergenic** |
| BE3 | #1 | BE3-insert 2 | chr12:102836616-102836617 | Btbd7 **Intronic** |
| BE3 | #2 | BE3-insert 3 | chr1:8155815-8155816 | Gm38216\|Gm38024 |
| BE3 | #2 | BE3-insert 4 | chr6:106085816-106085817 | Cntn4 **Intronic** |
| BE3 | #2 | BE3-insert 5 | chr7:122759154-122759155 | Cacng3 **Intronic** |
| BE3 | #2 | BE3-insert 2 | chr12:102836616-102836617 | Btbd7 **Intronic** |
| BE3 | #2 | BE3-insert 6 | chr18:87054755-87054756 | Gm5971\|Gm5234 **Intergenic** |
| BE3 | #3 | BE3-insert 7 | chr1:179249731-179249732 | Smyd3 **Intronic** |
| BE3 | #3 | BE3-insert 8 | chr7:44299731-44299732 | Gm18307\|Gm7238 **Intergenic** |
| BE3 | #3 | BE3-insert 2 | chr12:102836616-102836617 | Btbd7 **Intronic** |
| BE3 | #3 | BE3-insert 9 | chr13:54600579-54600580 | Cltb **Intronic** |
| YE1-BE3-FNLS | #1 | YE1-BE3-FNLS-insert 1 | chr2:143578196-143578197 | Pcsk2 **Intronic** |
| YE1-BE3-FNLS | #1 | YE1-BE3-FNLS-insert 2 | chr6:130690506-130690507 | Gm27013 **Intronic** |
| YE1-BE3-FNLS | #1 | YE1-BE3-FNLS-insert 3 | chr9:23270781-23270782 | Bmper **Intronic** |
| YE1-BE3-FNLS | #1 | YE1-BE3-FNLS-insert 4 | chr13:54600579-54600580 | Cltb **Intronic** |
| YE1-BE3-FNLS | #2 | YE1-BE3-FNLS-insert 1 | chr2:143578196-143578197 | Pcsk2 **Intronic** |
| YE1-BE3-FNLS | #2 | YE1-BE3-FNLS-insert 2 | chr6:130690506-130690507 | Gm27013 **Intronic** |
| YE1-BE3-FNLS | #2 | YE1-BE3-FNLS-insert 3 | chr9:23270781-23270782 | Bmper **Intronic** |
| YE1-BE3-FNLS | #2 | YE1-BE3-FNLS-insert 4 | chr13:54600579-54600580 | Cltb **Intronic** |
| YE1-BE3-FNLS | #3 | YE1-BE3-FNLS-insert 1 | chr2:143578196-143578197 | Pcsk2 **Intronic** |
| YE1-BE3-FNLS | #3 | YE1-BE3-FNLS-insert 2 | chr6:130690506-130690507 | Gm27013 **Intronic** |
| YE1-BE3-FNLS | #3 | YE1-BE3-FNLS-insert 3 | chr9:23270781-23270782 | Bmper **Intronic** |
| ABE7.10$^{F148A}$ | #1 | ABE7.10$^{F148A}$-insert 1 | chr3:139126962-139126963 | Rap1gds1\|Gm4862 **Intergenic** |
| ABE7.10$^{F148A}$ | #2 | ABE7.10$^{F148A}$-insert 1 | chr3:139126962-139126963 | Rap1gds1\|Gm4862 **Intergenic** |
| ABE7.10$^{F148A}$ | #2 | ABE7.10$^{F148A}$-insert 2 | chr5:89060403-89060404 | Slc4a4 **Intronic** |
| ABE7.10$^{F148A}$ | #2 | ABE7.10$^{F148A}$-insert 3 | chr18:4283951-4283952 | Gm10557\|Gm7400 **Intergenic** |
| ABE7.10$^{F148A}$ | #3 | ABE7.10F148A-insert 1 | chr3:139126962-139126963 | Rap1gds1\|Gm4862 **Intergenic** |
| ABE7.10$^{F148A}$ | #3 | ABE7.10F148A-insert 3 | chr18:4283951-4283952 | Gm10557\|Gm7400 **Intergenic** |

**Supplementary Table 3. sgRNA sequences and primers for Sanger sequencing.**

| Locus | Target sequences | Primers | Sequence |
|---|---|---|---|
| *Hpd* | caacccagaaggtcaccgag | Outer forward primer | gaaatgtcttaggaggttagcc |
| | | Outer reverse primer | ggttccaaagtgccagtcct |
| | | Inner forward primer | agatgggagcagggtgagtc |
| | | Inner reverse primer | ctcgcactagccaatcccag |
| *Dmd* | ataataggggacgaacaggg | Outer forward primer | ccattgcattctaggtcatgag |
| | | Outer reverse primer | ctgacaggaagcagcacaac |
| | | Inner forward primer | ggcctatagagttcctgtatcc |
| | | Inner reverse primer | cttatgcaagtaacctgctgtc |
| *Tyr* | acctcagttccccttcaaag | Outer forward primer | gtctgtgacactcattaacc |
| | | Outer reverse primer | aaatgtggctgctgaagtac |
| | | Inner forward primer | gtattgccttctgtggagtt |
| | | Inner reverse primer | ctgctgaagtaccagtcttt |

**Supplementary Table 4. Primers for targeted deep sequencing.**

| Locus | group | Primers | Sequence |
|---|---|---|---|
| *Hpd* | GFP-#1 | Forward primer | tgctcaacgaatgctgcccaccatggat |
| | | Reverse primer | gtggtaaaacttacagtttccgcagttg |
| | GFP-#2 | Forward primer | ctacgaaagaatgctgcccaccatggat |
| | | Reverse primer | gtggtaaaacttacagtttccgcagttg |
| | GFP-#3 | Forward primer | gtcaacccgaatgctgcccaccatggat |
| | | Reverse primer | gtggtaaaacttacagtttccgcagttg |
| | BE3-#1 | Forward primer | tagttatcgaatgctgcccaccatggat |
| | | Reverse primer | gtggtaaaacttacagtttccgcagttg |
| | BE3-#2 | Forward primer | gaaaaactgaatgctgcccaccatggat |
| | | Reverse primer | gtggtaaaacttacagtttccgcagttg |
| | BE3-#3 | Forward primer | gagcgtttgaatgctgcccaccatggat |
| | | Reverse primer | gtggtaaaacttacagtttccgcagttg |
| | YE1-BE3-FNLS-#1 | Forward primer | tcgctgaagaatgctgcccaccatggat |
| | | Reverse primer | gacacaagacttacagtttccgcagttg |
| | YE1-BE3-FNLS-#2 | Forward primer | gaaaaactgaatgctgcccaccatggat |
| | | Reverse primer | gacacaagacttacagtttccgcagttg |
| | YE1-BE3-FNLS-#3 | Forward primer | gagcgtttgaatgctgcccaccatggat |
| | | Reverse primer | gacacaagacttacagtttccgcagttg |
| *Dmd* | GFP-#1 | Forward primer | atcacgagggccctcttgccccttaac |
| | | Reverse primer | acagtggaatgcagagttaatacacacg |
| | GFP-#2 | Forward primer | cgatgtagggccctcttgccccttaac |
| | | Reverse primer | acagtggaatgcagagttaatacacacg |
| | GFP-#3 | Forward primer | ttaggcagggccctcttgccccttaac |
| | | Reverse primer | acagtggaatgcagagttaatacacacg |
| | ABE7.10$^{F148A}$-#1 | Forward primer | atcacgagggccctcttgccccttaac |
| | | Reverse primer | gccaatgaatgcagagttaatacacacg |
| | ABE7.10$^{F148A}$-#2 | Forward primer | cgatgtagggccctcttgccccttaac |
| | | Reverse primer | gccaatgaatgcagagttaatacacacg |
| | ABE7.10$^{F148A}$-#4 | Forward primer | tgaccaagggccctcttgccccttaac |
| | | Reverse primer | gccaatgaatgcagagttaatacacacg |

**Supplementary Table 5. Primers for verification of random selected RNA SNVs in BE3 muscle.**

| Locus | Primers | Sequence |
|---|---|---|
| NC_000067.6-93508362 | Outer forward primer | atagctggcctgactaatca |
| | Outer reverse primer | tcactgaaaaggggaagagt |
| | Inner forward primer | tgcttactagaattgaagtcaca |
| | Inner reverse primer | caaccctgaaatcctgcttt |
| NC_000070.6-15285798 | Outer forward primer | gtattcccaaggtaccagga |
| | Outer reverse primer | ttaacaggtgtgatgctgtg |
| | Inner forward primer | ccacctagttttcaatctggt |
| | Inner reverse primer | tccaacaatgagacttgataaca |
| NC_000076.6-33367128 | Outer forward primer | tgggtctcagtgataagctt |
| | Outer reverse primer | agccttttgtagttcccaaa |
| | Inner forward primer | tgtgaagctaaaggaatggtc |
| | Inner reverse primer | gttcttacttaacttatgtgcagt |
| NC_000082.6-64766215 | Outer forward primer | gagcagcggattttgagata |
| | Outer reverse primer | aaaatgtgcgtcttctcctc |
| | Inner forward primer | ggaccgcaataagttctcag |
| | Inner reverse primer | aagaagctgcagaaacaaca |
| NC_000086.7-9654816 | Outer forward primer | tgagccttcaacaacaacaa |
| | Outer reverse primer | aggtacctatcggatgtgtg |
| | Inner forward primer | gactgactttgcatttggtg |
| | Inner reverse primer | atgtagagttaggacaaatggg |
| NC_000068.7-119602997 | Outer forward primer | catctgacttgggtgttgag |
| | Outer reverse primer | ctgggaccctgcatatctaa |
| | Inner forward primer | aagtagttgctttgttctgaaaa |
| | Inner reverse primer | cggcaggactctatttctaaa |
| NC_000073.6-131445809 | Outer forward primer | gtgaagccattgatcagtgt |
| | Outer reverse primer | gatccaacagccatgaactt |
| | Inner forward primer | tttagaccttgaatccaaagaga |
| | Inner reverse primer | ttacaggagtggaaggtgtc |
| NC_000076.6-61429145 | Outer forward primer | ccagagaaacaaaggggaag |
| | Outer reverse primer | gtgacaggtcctcagttttc |
| | Inner forward primer | attcatgggagtctgggtc |
| | Inner reverse primer | gcctgctcagctctttatag |
| NC_000086.7-159386968 | Outer forward primer | cttgagaggaaatgaggggа |
| | Outer reverse primer | caaaccgaggagatgaagtc |
| | Inner forward primer | ggatcttgtcgtctggtttt |
| | Inner reverse primer | cagcatgtctagtgagttcc |
| NC_000068.7-120507290 | Outer forward primer | cctttccttctccactctct |
| | Outer reverse primer | acagtctgaggctacacttt |
| | Inner forward primer | gcttcctcctttccttccta |
| | Inner reverse primer | agtggggttgaacagtaagt |
| NC_000071.6-20759239 | Outer forward primer | tcctagaactctactgctgc |
| | Outer reverse primer | gccatgtgttaaatgttgcc |
| | Inner forward primer | gatacaacaatctcactttattcct |
| | Inner reverse primer | ggctttgtctgatatgttcct |
| NC_000074.6-124909354 | Outer forward primer | caacaatgccaaacagttcc |
| | Outer reverse primer | cgtgaatgtggtatgtggtt |
| | Inner forward primer | cggctctacttggttgtttt |
| | Inner reverse primer | gccaaaatgtgtgtatgcag |

**Supplementary Table 6. Insert locations of BE3 in BE3 mice with normal weight and obesity.**

| Group | | Integration name | Integration site | Location |
|---|---|---|---|---|
| BE3 mice with normal weight | #1 | BE3-insert 10 | chr1:69683654-69683655 | Ikzf2 **Intronic** |
| | | BE3-insert 11 | chr9:37485434-37485435 | Gm47945\|Gm47963 **Intergenic** |
| | | BE3-insert 1 | chr11:23139312-23139313 | Gm12059\|1700061J23Rik **Intergenic** |
| | | BE3-insert 12 | chr11:58169177-58169178 | Gemin5\|Mrpl22 **Intergenic** |
| | #2 | BE3-insert 13 | chr5:148296121-148296122 | Mtus2 **Intronic** |
| | | BE3-insert 4 | chr6:106085816-106085817 | Cntn4 **Intronic** |
| | | BE3-insert 8 | chr7:44299731-44299732 | Gm18307\|Gm7238 **Intergenic** |
| | | BE3-insert 14 | chr10:92916728-92916729 | Cfap54 **Intronic** |
| | | BE3-insert 1 | chr11:23139312-23139313 | Gm12059\|1700061J23Rik **Intergenic** |
| | | BE3-insert 12 | chr11:58169177-58169178 | Gemin5\|Mrpl22 **Intergenic** |
| | #3 | BE3-insert 15 | chr6:37202544-37202545 | Dgki **Intronic** |
| | | BE3-insert 1 | chr11:23139312-23139313 | Gm12059\|1700061J23Rik **Intergenic** |
| | | BE3-insert 16 | chr11:30464035-30464036 | 4930505A04Rik **Intronic** |
| | | BE3-insert 12 | chr11:58169177-58169178 | Gemin5\|Mrpl22 **Intergenic** |
| | | BE3-insert 17 | chr15:101114617-101114618 | Fignl2\|Ankrd33 **Intergenic** |
| BE3 mice with obesity | #1 | BE3-insert 13 | chr5:148296121-148296122 | Mtus2 **Intronic** |
| | | BE3-insert 4 | chr6:106085816-106085817 | Cntn4 **Intronic** |
| | | BE3-insert 8 | chr7:44299731-44299732 | Gm18307\|Gm7238 **Intergenic** |
| | | BE3-insert 11 | chr9:37485434-37485435 | Gm47945\|Gm47963 **Intergenic** |
| | | BE3-insert 1 | chr11:23139312-23139313 | Gm12059\|1700061J23Rik **Intergenic** |
| | #2 | BE3-insert 13 | chr5:148296121-148296122 | Mtus2 **Intronic** |
| | | BE3-insert 4 | chr6:106085816-106085817 | Cntn4 **Intronic** |
| | | BE3-insert 8 | chr7:44299731-44299732 | Gm18307\|Gm7238 **Intergenic** |
| | | BE3-insert 11 | chr9:37485434-37485435 | Gm47945\|Gm47963 **Intergenic** |
| | | BE3-insert 1 | chr11:23139312-23139313 | Gm12059\|1700061J23Rik **Intergenic** |
| | #3 | BE3-insert 13 | chr5:148296121-148296122 | Mtus2 **Intronic** |
| | | BE3-insert 8 | chr7:44299731-44299732 | Gm18307\|Gm7238 **Intergenic** |
| | | BE3-insert 1 | chr11:23139312-23139313 | Gm12059\|1700061J23Rik **Intergenic** |

**Supplementary Table 7. Primes and probes for droplet digital PCR reactions.**

| Gene | Primers | Sequence |
|---|---|---|
| N-YE1-BE3-FNLS | Primer F | gtatccacggagtcccagcag |
| | Primer R | cgtaaagcaggcaggtctcc |
| | *Probe* | 5’VIC/ggcggatcgagccccatgag/BHQ-1 |
| C-YE1-BE3-FNLS | Primer F | gtcgaagagaatcccgggcca |
| | Primer R | cgccggacacgctgaacttg |
| | *Probe* | 5’VIC/ccatcctggtcgagctggacggcg/BHQ-1 |
| Sod2 (Reference) | Primer F | aaggagcaaggtcgcttaca |
| | Primer R | ggcactgacatgggaagagt |
| | *Probe* | 5’6-FAM/caggacccattgcaaggaaca/BHQ-1 |
