# Supplementary material for: Cytosine base editors induce off-target mutations and adverse phenotypic effects in transgenic mice
Source: Nat Commun. 2023 Mar 30;14:1784. doi: 10.1038/s41467-023-37508-7 (PMC10063651; doi:10.1038/s41467-023-37508-7)
Supplement: Supplementary file 2 — Reporting Summary [file 41467_2023_37508_MOESM2_ESM.pdf]

nature portfolio

Corresponding author(s): Erwei Zuo

Last updated by author(s): Marth 13, 2023

Reporting Summary

Nature Portfolio wishes to improve the reproducibility of the work that we publish. This form provides structure for consistency and transparency in reporting. For further information on Nature Portfolio policies, see our Editorial Policies and the Editorial Policy Checklist.

Statistics

For all statistical analyses, confirm that the following items are present in the figure legend, table legend, main text, or Methods section.

n/a	Confirmed	
☐	☒	The exact sample size (*n*) for each experimental group/condition, given as a discrete number and unit of measurement
☐	☒	A statement on whether measurements were taken from distinct samples or whether the same sample was measured repeatedly
☐	☒	The statistical test(s) used AND whether they are one- or two-sided<br>*Only common tests should be described solely by name; describe more complex techniques in the Methods section.*
☒	☐	A description of all covariates tested
☒	☐	A description of any assumptions or corrections, such as tests of normality and adjustment for multiple comparisons
☐	☒	A full description of the statistical parameters including central tendency (e.g. means) or other basic estimates (e.g. regression coefficient) AND variation (e.g. standard deviation) or associated estimates of uncertainty (e.g. confidence intervals)
☐	☒	For null hypothesis testing, the test statistic (e.g. *F*, *t*, *r*) with confidence intervals, effect sizes, degrees of freedom and *P* value noted<br>*Give P values as exact values whenever suitable.*
☒	☐	For Bayesian analysis, information on the choice of priors and Markov chain Monte Carlo settings
☒	☐	For hierarchical and complex designs, identification of the appropriate level for tests and full reporting of outcomes
☒	☐	Estimates of effect sizes (e.g. Cohen's *d*, Pearson's *r*), indicating how they were calculated

Our web collection on statistics for biologists contains articles on many of the points above.

Software and code

Policy information about availability of computer code

Data collection	Illumina Novaseq 6000, Summit Software (5.2)
Data analysis	FastQC (v0.11.8), Trimmomatic (v0.39), STAR (v2.1.7), Picard (v2.5.7.0), GATK (v4.1.5), annovar (v2020-06-08), HTseq (v0.11.3), CRISPResso2 (v2.0.32), SOAPnupk (v2.1.6), BWA mem (v0.7.12), Mutect2 (v4.1.5), Lofreq (v2.1.5), Strelka (v2.7.1), Scalpel (v0.5.4), Manta (v1.6.0), Lumpy (v0.2.13), Delly (v0.7.6), Flow Jo (v10.0.7). All softwares used in this manuscripts were described in the methods.

For manuscripts utilizing custom algorithms or software that are central to the research but not yet described in published literature, software must be made available to editors and reviewers. We strongly encourage code deposition in a community repository (e.g. GitHub). See the Nature Portfolio guidelines for submitting code & software for further information.

Data

Policy information about availability of data

All manuscripts must include a data availability statement. This statement should provide the following information, where applicable:

- Accession codes, unique identifiers, or web links for publicly available datasets
- A description of any restrictions on data availability
- For clinical datasets or third party data, please ensure that the statement adheres to our policy

The raw data were deposited in the National Center for Biotechnology Information (NCBI) Sequence Read Archive database under accession code PRJNA831302 (https://www.ncbi.nlm.nih.gov/bioproject/PRJNA831302). All raw sequence data are also available in the China National GeneBank DataBase (CNGBdb) with the accession number CNP0002932 (https://db.cngb.org/search/project/CNP0002932/).

Human research participants

Policy information about studies involving human research participants and Sex and Gender in Research.

Reporting on sex and gender	N/A.
Population characteristics	N/A.
Recruitment	N/A.
Ethics oversight	N/A.

Note that full information on the approval of the study protocol must also be provided in the manuscript.

Field-specific reporting

Please select the one below that is the best fit for your research. If you are not sure, read the appropriate sections before making your selection.

☒ Life sciences ☐ Behavioural & social sciences ☐ Ecological, evolutionary & environmental sciences

For a reference copy of the document with all sections, see nature.com/documents/nr-reporting-summary-flat.pdf

Life sciences study design

All studies must disclose on these points even when the disclosure is negative.

Sample size	Sample size is indicated on the Figure Legends. No statistical method was used to predetermine sample size. Sample size was chosen based on our previous works (Zuo et al., Nature Methods, 2020; Zuo et al., Science, 2019; Zhou et al., Nature, 2019).
Data exclusions	No data was excluded.
Replication	Independent biological replicates were sampled on different days since we selected mice of the same age for each experiments. All statistic data are presented as mean ± standard error of the mean. All samples represent a minimum of two replicates. All attempts at replication were successful.
Randomization	Randomization was not used in this study. In all animal studies, mice were grouped by genotype and matched by age and sex.
Blinding	Blinding of samples was not performed in this study since our samples had obvious phenotypes depending on the genotype and the results were consistent.

Reporting for specific materials, systems and methods

We require information from authors about some types of materials, experimental systems and methods used in many studies. Here, indicate whether each material, system or method listed is relevant to your study. If you are not sure if a list item applies to your research, read the appropriate section before selecting a response.

Materials & experimental systems

n/a	Involved in the study
☐	☒ Antibodies
☐	☒ Eukaryotic cell lines
☒	☐ Palaeontology and archaeology
☐	☒ Animals and other organisms
☒	☐ Clinical data
☒	☐ Dual use research of concern

Methods

n/a	Involved in the study
☒	☐ ChIP-seq
☐	☒ Flow cytometry
☒	☐ MRI-based neuroimaging

Antibodies

Antibodies used	Cas9 (7A9-3A3) Mouse mAb (14697S, Cell Signaling Technology, 1:1000), HRP-conjugated Beta Actin (2D4H5) Monoclonal antibody (HRP-66009, Proteintech, 1:5000), HRP-conjugated Affinipure Goat Anti-Mouse IgG(H+L) (SA00001-1, Proteintech, 1:5000)
Validation	Cas9 (7A9-3A3) Mouse mAb (14697S; Cell Signaling Technology) (Specificity were confirmed in this study and other studies (PMID: 32796846; PMID: 32843625; PMID: 33547076; PMID: 33662274; PMID: 33432198), by manufacturer (https://www.cellsignal.com/products/primary-antibodies/cas9-7a9-3a3-mouse-mab/14697)).<br>HRP-conjugated Beta Actin (2D4H5) Monoclonal antibody (HRP-66009; Proteintech) (Specificity were confirmed in this study and other studies (PMID: 33397955; PMID: 33859191; PMID: 34783310; PMID: 34522852), by manufacturer (https://www.ptgcn.com/products/beta-Actin-Antibody-HRP-66009.htm)).<br>HRP-conjugated Affinipure Goat Anti-Mouse IgG(H+L) (SA00001-1; Proteintech) (Specificity were confirmed in this study and other studies (PMID: 30962431; PMID: 27053551; PMID: 29931370; PMID: 29665050), by manufacturer (https://www.ptglab.com/products/HRP-conjugated-Affinipure-Goat-Anti-Mouse-IgG-H-L-secondary-antibody.htm)).

Eukaryotic cell lines

Policy information about cell lines and Sex and Gender in Research

Cell line source(s)	In this study, we only used one cell line HEK293T for AAVs production, which was from the Cell bank of Shanghai Institute of Biochemistry and Cell Biology, Chinese Academy of Sciences.
Authentication	Cell lines were authenticated with STR profiling by supplier
Mycoplasma contamination	Cells used in this study were free of mycoplasma contamination. Mycoplasma contamination was determined by PCR the supernatant of HEK293T cells.
Commonly misidentified lines<br>(See ICLAC register)	None of the cell lines used was listed in the database of ICLAC.

Animals and other research organisms

Policy information about studies involving animals; ARRIVE guidelines recommended for reporting animal research, and Sex and Gender in Research

Laboratory animals	Two mouse strains were used for generation of the transgenic mice in the manuscript: C57BL/6J mice, female, 3-4 week-old; ICR mice, females, 8 week-old. Transgenic mice of both sexes and various ages were used in this study. Detailed animal information for each experiment can be found in methods.<br>Housing conditions were: 12-hour dark/light cycle lights on at 8 am, temperature 22 °C, humidity 30-70%.
Wild animals	No wild animals were used.
Reporting on sex	We performed body weight measurements disaggregated for sex because of the weight difference between males and females, as described in methods.
Field-collected samples	No field-collected samples were used.
Ethics oversight	All animal procedures were reviewed and approved by the Life Sciences Ethics Committee of Agricultural Genomics Institute at Shenzhen, Chinese Academy of Agricultural Sciences, and performed in compliance with all relevant ethical regulations.

Note that full information on the approval of the study protocol must also be provided in the manuscript.

Flow Cytometry

Plots

Confirm that:

- [x] The axis labels state the marker and fluorochrome used (e.g. CD4-FITC).
- [x] The axis scales are clearly visible. Include numbers along axes only for bottom left plot of group (a 'group' is an analysis of identical markers).
- [x] All plots are contour plots with outliers or pseudocolor plots.
- [x] A numerical value for number of cells or percentage (with statistics) is provided.

Methodology

Sample preparation	Primary hepatocytes were isolated by standard two-step collagenase perfusion method and purified by 40% Percoll (Sigma) at low-speed centrifugation (1,000 rpm, 10 min), GFP and tdTomato-positive hepatocytes were isolated by Flow Cytometry.
Instrument	MoFlo XDP (Beckman)
Software	Collect: Summit Software version 5.2<br>Analyze: Flow Jo (v10.0.7).
Cell population abundance	20000 cells were sorted per group. Positive and negative boundaries were determined by control cells that were not infected with any plasmids.
Gating strategy	Positive and negative boundaries were determined by control cells that were not infected with any plasmids.

- [x] Tick this box to confirm that a figure exemplifying the gating strategy is provided in the Supplementary Information.
