# Supplementary material for: Cytosine base editors induce off-target mutations and adverse phenotypic effects in transgenic mice
Source: Nat Commun. 2023 Mar 30;14:1784. doi: 10.1038/s41467-023-37508-7 (PMC10063651; doi:10.1038/s41467-023-37508-7)
Supplement: Supplementary file 3 — Description of Additional Supplementary Files [file 41467_2023_37508_MOESM3_ESM.pdf]

Title: Supplementary Data 1

Description: *P* values for WT, BE3, YE1-BE3-FNLS or ABE7.10^{F148A} group compared to GFP group from birth to 66 weeks.

Title: Supplementary Data 2

Description: Deep sequencing of primes for identification of integration sites.

Title: Supplementary Data 3

Description: PCR primes for confirmation of integration sites.
